# Alternative H_2_O_2_ Production Processes: An Outlook on Candidate Technologies Beyond the Anthraquinone Process

**DOI:** 10.1021/acsomega.5c07503

**Published:** 2025-12-10

**Authors:** Stavros-Alexandros Theofanidis, Amvrosios G. Georgiadis, Christianus J. W. Hop, Xiaobin Yu, Vasileia-Loukia Yfanti, Guillaume Fayet, Claire Villemur, Hank Vleeming, Evangelos Delikonstantis, Richard H. Heyn

**Affiliations:** † AristEng S.à r.l., 20, Avenue Pasteur, Luxembourg City L-2310, Luxembourg; ‡ Process Design Center BV, Paardeweide 7, Breda NL-4824EH, The Netherlands; § Ineris, Parc Technologique ALATA, BP 2, Verneuil-en-Halatte 60550, France; ∥ SINTEF Industry, P.O. Box 124 Blindern, Oslo 0314, Norway

## Abstract

Hydrogen peroxide is currently made on a large scale via the auto-oxidation (AO) process, an indirect process via the hydrogenation of anthraquinone over a Pd catalyst, followed by reaction with O_2_. The AO process has a significant climate footprint, since H_2_ is usually sourced from methane steam reforming. A more sustainable H_2_O_2_ production process is, therefore, highly relevant. Potential alternative routes include the direct reaction of H_2_ and O_2_ and processes that use electricity, either directly via electrochemical or bioelectrochemical systems or indirectly via plasmas. Despite research on these routes, there are currently no real challengers to the established AO process. An overview of these alternative processes is provided before considering the energetic, techno-economic, life cycle, and safety challenges facing them. By highlighting these challenges through comparison with the AO process, this review aims to inspire further work for scaling up these alternative processes for truly determining their industrial viability. As these processes may ultimately prove more advantageous than the AO process for on-demand production, their industrialization might not need to occur on the same production scale as that of the AO process.

## Graphical abstract

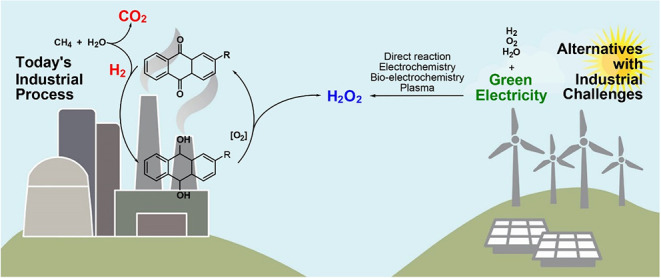

## Introduction

Hydrogen peroxide, H_2_O_2_, is a versatile chemical that is used in a variety of applications, primarily because of its strong oxidation potential and benign products, as it releases only H_2_O and O_2_ upon use. H_2_O_2_ is used as a chemical reagent (e.g., epoxidation, production of other oxidizers), for the bleaching of cellulosic materials (e.g., cotton and wood) and for disinfection and cleaning.^1^ European industry uses 50% of the produced H_2_O_2_ in the chemical and pharmaceutical sectors, 41% in the pulp and paper sector, and 8% in the textiles sector.^2^ The volume of the global H_2_O_2_ market in 2023 was estimated to be 4.3 million tons (Mt) with a value of USD 1.82 billion, with an anticipated compound annual growth rate of 3.8% from 2023 to 2032.^3^

The first industrial H_2_O_2_ process, the Weissenstein process, was electrochemical and was established in 1908. In this process, H_2_SO_4_ was oxidized to peroxodisulfuric acid, HO_3_SOOSO_3_H, and then hydrolyzed to H_2_O_2_.^4^ Improvements to this process evolved from the use of sulfate salts. For example, the Löwenstein and Laporte processes used (NH_4_)_2_SO_4_ rather than H_2_SO_4_, while the Pietzsch–Adolph process precipitated the intermediate ammonium peroxydisulfate, (NH_4_)_2_(O_3_SOOSO_3_), as a potassium salt that was then isolated, acidified, and hydrolyzed.^4^ These electrochemical processes were gradually replaced by the Riedl–Pfleiderer, anthraquinone, or auto-oxidation (AO) process, the first plant of which was commissioned in 1953. The AO process is now the global standard for industrial H_2_O_2_ production. While the change from an electrochemical to the thermochemical AO process was undoubtedly driven by the unfavorable economics of high capital expenditures, electricity consumption, and the required purity of chemicals and electrolytes, the original electrochemical processes would also no longer meet today’s health and safety considerations. For example, it is reported that the cathode in the Pietzsch–Adolph process was wrapped with asbestos rope to eliminate the need for a catholyte and that the produced ammonia and hydrogen gases were vented directly to the atmosphere, requiring well-ventilated cell rooms.^4^

The AO process accounts for 95% of the worldwide H_2_O_2_ production and is an indirect synthesis starting with the hydrogenation of an alkylanthraquinone over a Pd catalyst, separation of the hydroquinone, and finally oxidation with O_2_ (air) back to the anthraquinone, producing H_2_O_2_ quantitatively. A whole host of solvents, including alkylated benzenes, alkyl phosphates, tetraalkyl ureas, and other organics, are required to keep all of the components in solution and facilitate the reaction and separation steps in the process.^5^ Paradoxically, although H_2_O_2_ is considered one of the greenest oxidizing agents^6^ and is not a carbon-containing chemical, fossil carbon is one of the key reagents in the AO production process, since the required H_2_ is generated from steam methane reforming (SMR). This, together with a total energy consumption of 2.9–5.7 GJ/1000 kg_H2O2_,^7^ is the main reason that the CO_2‑equiv_ emissions of the AO process exceed 2.8 Mt annually (1.33 kg_CO2_/kg_H2O2_).^8,9^

Electrification has been identified as one of the main routes to reducing the CO_2_ footprint of the chemical industry,^10^ particularly when combined with efforts to enhance the green electricity supply,^11^ i.e., renewable energy sources such as wind, solar, and hydropower.^12^ Of the different electrification strategies, electrochemistry can beneficially lower reaction temperatures, reducing thermal losses, decreasing material stability issues, and overcoming equilibrium conversion limitations in exothermic reactions.^13^ However, a challenge associated with electrification is the increased electricity demand that will accompany the transition of the chemical industry from conventional fossil-fuel-based to electrified routes. One estimate suggests that renewable electricity demand will increase 10-fold by 2050.^14^ Fortunately, electrification also allows the operational flexibility necessary for smaller, modular facilities that can be located in regions where renewable electricity is abundant^14^ and that can be more easily integrated into distributed power grids. Electrochemical processes can thus provide grid-balancing services that can be economically advantageous.^13^ Indeed, because of the energy demands of the chemical industry, collaboration and integration between the chemical and energy sectors are necessary to achieve emissions reductions through the electrification of the chemical industry.^12^ Furthermore, if an electrified chemical process could make easily stored products, then the process could be run when sustainable electricity is cheap and available, provided that the cost savings outbalance additional investments.

While the hydrogen atoms in H_2_O_2_ and a reduced CO_2_ footprint could come via a modified AO process using green H_2_ produced from H_2_O electrolysis, the direct use of electricity has the potential to be more efficient. A number of alternative syntheses of H_2_O_2_ are therefore under investigation.^9,15,16^ For such syntheses, there is no need for anthraquinone, a costly component that adds approximately USD 2 billion annually to the expenses of H_2_O_2_ manufacturers.^8^ Despite the potential for improved economics, there are still no examples of an alternative industrial process for producing the high-concentration H_2_O_2_ solutions (>30%) required for most applications. A frequently cited example of an active alternative process is the Headwaters–Evonik joint venture in Ulsan, Korea, where H_2_O_2_ was proposed to be produced by the direct reaction of H_2_ and O_2_ and supplied directly to a neighboring propylene oxide facility (the Hydrogen Peroxide to Propylene Oxide process).^17^ However, the implementation of the technology was referred to as a “future target” by the joint venture, without any recent reference about the progress in meeting this target. Although the Ulsan facility has more than doubled its capacity to over 68,000 t/yr since its acquisition from Kemira Oyi in 2006, there is no definitive public evidence confirming the industrial implementation of an alternative H_2_O_2_ synthesis at this site.^18–20^ An alternative H_2_O_2_ production method has been commercialized by the Danish company HPNow, providing modular, electrochemical H_2_O_2_ production units primarily for water treatment. While the inputs are only H_2_O and air, the limitation is that the obtained H_2_O_2_ concentrations of 0.25–0.5% are not relevant for the textiles and pulp and paper industries. Cost is another challenge.^21^

Environmental pressures thus suggest the need for industrial H_2_O_2_ production to complete the circle, starting from the initial electrochemical routes in the early to mid-1900s to the currently dominant, fossil-fuel-driven AO production process, and now back to an electricity-driven process. The direct use of electricity from renewable energy sources could offer a sustainable pathway for the industrial production of H_2_O_2_. However, the alternative H_2_O_2_ production technologies still suffer from limitations that have prevented them from challenging the supremacy of the AO process. After a brief overview of the current state-of-play of four alternative processes for H_2_O_2_ production, thermochemical, electrochemical, bioelectrochemical, and plasmatic, the main economic, environmental, and safety challenges of these alternative processes are critically reviewed and compared against the AO process. The aim is to highlight the deficiencies that currently hamper the market penetration of these alternative technologies and thus provide impetus to address them. An overview of the alternative processes is shown in Figure 1.

**Figure 1:**
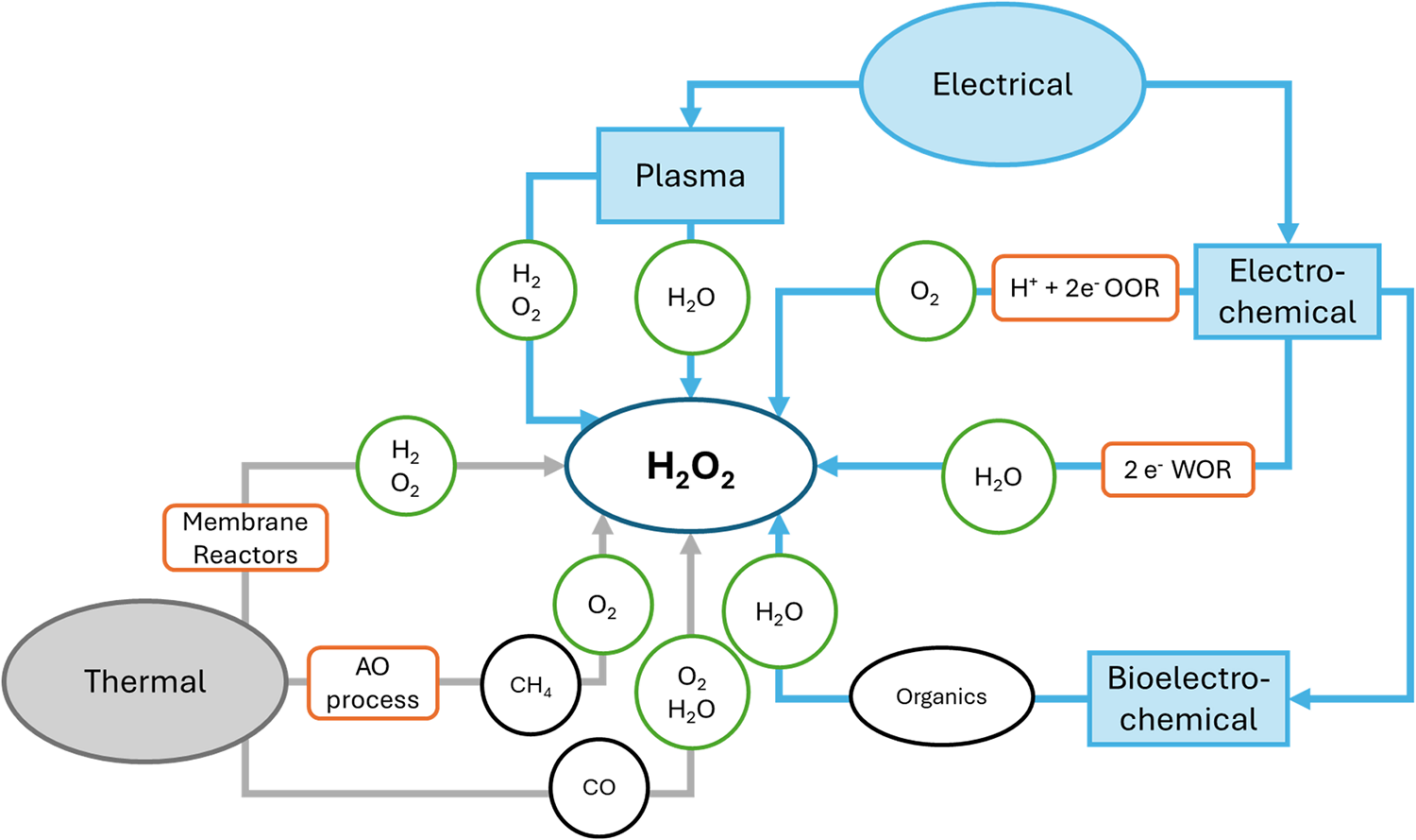
Overview of alternative H_2_O_2_ production processes. Gray, thermal; blue, electrical; orange boxes, technologies; green circles, sustainable reactants; and black circles, auxiliary reactants.

## Alternative Technologies for H_2_O_2_ Production

### Thermochemical

Thermochemical processes, specifically the direct synthesis of H_2_O_2_ (DSHP) and the synthesis from CO/O_2_/H_2_O mixtures, offer promising alternatives for H_2_O_2_ production. DSHP involves the catalytic reaction of H_2_ and O_2_ to form H_2_O_2_, offering a simplified, one-step process. However, challenges such as the explosive nature of H_2_/O_2_ mixtures and the competitive formation of H_2_O necessitate stringent safety measures and catalyst optimization. On the other hand, synthesis from CO/O_2_/H_2_O mixtures leverages the in situ formation of H_2_O_2_ via intermediate species, providing improved control over reaction conditions and potentially mitigating safety concerns. The primary distinction lies in the reactants and their implications for process safety and scalability, with the CO-based method offering enhanced versatility under milder reaction conditions.

While a one-step DSHP (eq 1) could simplify H_2_O_2_ production, this reaction is complex, with exothermic and thermodynamically favorable side reactions (eqs 2–4). Reaction pathways depend on the catalyst, reaction medium additives, and conditions.^6,22^ Despite initial findings in 1914 ^23^ and multiple patents,^6^ industrial-scale implementation remains elusive. The main challenges of DSHP include managing the explosive H_2_/O_2_ mixture (*vida infra*) and achieving higher selectivity toward H_2_O_2_ instead of H_2_O, as common catalysts also promote H_2_ combustion and H_2_O_2_ decomposition.^24^ Due to H_2_O_2_ instability, DSHP needs a solid catalyst and a H_2_O/MeOH solvent to dissolve H_2_ and O_2_. To address mass transfer barriers, microbubbles of H_2_ and O_2_ have been suggested.^25^ Viable H_2_O_2_ concentrations have been achieved with a decane-1-ol/H_2_O solvent,^26^ and proton–electron transfer can enhance H_2_O_2_ production in MeOH/H_2_O.^27^ However, these solutions do not fully mitigate explosion risks associated with high catalyst activity.^28^

1 $$H_{2}O_{2}\;\mathrm{formation}:\;H_{2}+O_{2}\to H_{2}O_{2};\;\Delta H=-135.8\;\mathrm{kJ}/\mathrm{mol};\;\Delta G^{o}=-120.4\;\mathrm{kJ}/\mathrm{mol}$$

2 $${\mathrm{direct}\;\mathrm{oxidation}\;\mathrm{to}\;H}_{2}O:\;H_{2}+0.5\;O_{2}\to H_{2}O;\;\Delta H=-241.6\;\mathrm{kJ}/\mathrm{mol};\;\Delta G^{o}=-237.2\;\mathrm{kJ}/\mathrm{mol}$$

3 $$H_{2}O_{2}\;\mathrm{decomposition}:\;H_{2}O_{2}\to H_{2}O+0.5O_{2};\;\Delta H=-105.8\;\mathrm{kJ}/mol;\;\Delta G^{o}=-116.8\;\mathrm{kJ}/mol$$

$$H_{2}O_{2}\;\mathrm{hydrogenation}:\;H_{2}O_{2}+H_{2}\to 2\;H_{2}O;\;\Delta H=-211.5\;\mathrm{kJ}/\mathrm{mol};\;\Delta G^{o}=-345.0\;\mathrm{kJ}/\mathrm{mol}$$ 4

In DSHP, the solvent plays a crucial role in facilitating the reaction. Acidic additives, such as phosphoric acid, stabilize H_2_O_2_ by lowering the pH and mitigating decomposition in the presence of basic impurities or reactive surfaces.^29^ HCl and HBr further enhance H_2_O_2_ selectivity by providing both acidic and anionic effects, which suppress side reactions like decomposition and overhydrogenation. However, the highly acidic conditions, combined with halides, necessitate the use of corrosion-resistant materials.^30^

Most catalysts for DSHP rely on noble metals supported on materials such as alumina, silica, and carbon (see Table 1).^24^ Palladium is often used in combination with supports designed to reduce corrosion or enhance stability, such as tungsten oxide on zirconia or functionalized carbons.^31,32^ Hydrophobic supports, like fluorine-treated carbon, reduce H_2_O_2_ side reactions by improving mass transport and preventing degradation.^31^ Advanced designs include Pd catalysts anchored on sulfonic acid resins, which offer high yields at moderate temperatures (40 °C) in MeOH.^29^ Alloying palladium with gold or platinum further enhances selectivity and stability, as evidenced by trimetallic Pt–Au–Pd catalysts that suppress H_2_O_2_ decomposition and increase overall yields.^33^ However, challenges persist, particularly at high H_2_O_2_ concentrations, where side reactions and catalyst degradation are exacerbated. Efforts to address these challenges focus on optimizing catalyst composition and structure, improving recovery and reuse of noble metals, and enhancing long-term stability under industrial conditions.^22^ H_2_O_2_ concentrations up to 10 wt % are achievable via DSHP but are limited by safety concerns and the high costs of noble metal catalysts, constraining broader industrial application.

**Table 1:** H_2_O_2_ Direct Synthesis with Noble Metal Catalysts

**catalyst**	**reactor type**	**selectivity (%)**	**formation rate** ^a^	**H**_**2**_ **O**_**2**_ **wt %**	***T*/bar/*t* (°C/bar/h)** ^b^
5% Pd@TiO_2_ ^34^	batch	70	194		27/89/0.28
0.4Au1.1Pd@TiNT^c^ ^35^	batch		174.9	0.14	5/20/0.67
Pd@XC72^d^ ^36^	batch	74	129	1.7	10/1/4
Pd/NPCs-PSS^e^ ^37^	batch	71.9	328.4	0.18	0/1/3
Pd_0.3/NPCS^f^ ^38^	batch	76.7	219		0/1/1
Pd(2%)/S-AC^g^ ^39^	batch	87	42.1		2/30/1
Pd(0.6%)/HHDMA^h^/AC^40^	batch	80	50.4		0/40/0.5
Pd_1.8%_873^i^ ^41^	batch	93	248		r.t./30/0.33
0.2Pt0.2Au4.6Pd^33^	batch		184		2/14/0.5
2.5Au2.5Pd^42^	batch		252	0.5	2/29/0.5
AuPd@SZ^j^ ^43^	batch	55	13.6		r.t./1/5
1Au3Pd^44^	batch	56.6	1141	0.12	0/38/3
AuPd^45^	batch	60.3	988.8	5.8	10/10/30
AuPd^46^	batch	99	426.7	0.12	5/20/1
Co_*x*_ Pd/C^47^	batch	88.1	83		2/30/1
1.0Au2.0Pd^48^	semibatch	48	69.9		10/1/n.a.
Au(2.2%)@hollowSiO_2_ ^49^	semibatch	15	24.8		10/1/n.a.
Pd(5.6%)/s-TiO_2_ ^50^	semibatch	21	85.7		30/n.a. /n.a.
Pd(4.1%)Sn/s-TiO_2_/H_2_ SO_4_ ^50^	semibatch	77	378.9		30/n.a. /n.a.
Pd_50_ Te_1_/Al_2_ O_3_ ^51^	semibatch	52.9	84.7		10/1/n.a.
Pd_1_ Au_0.7_/TiO_2_ ^52^	continuous	86	36	2.9	23/10/n.a.
Pd/Al_2_ O_3_ ^53^	continuous		2.8	0.388	25/1/n.a.

a The H_2_O_2_ production rates have been converted to mmol/g_cat_·h from the original sources.

b “r.t.” = room temperature; “n.a.” = not applicable.

c Nanotubes.

d A carbon black.

e Nitrogen-doped porous carbons stabilized by poly­(styrenesulfonate).

f N-doped porous carbon sphere.

g Sulfonate activated carbon.

h Hexadecylhydroxydimethylammonium.

i N-containing carbon nanotubes heated to 873 K.

j Sulfonated zirconia.

Membrane catalysts offer a safer method for DSHP by keeping H_2_ and O_2_ separated, thus mitigating H_2_/O_2_ mixture explosion concerns.^54^ Dense palladium membranes are particularly promising, enabling liquid-phase H_2_O_2_ synthesis by supplying atomic hydrogen to a catalyst layer on the membrane’s surface. A Pd/Ag alloy membrane on α- or γ-Al_2_O_3_ substrates facilitates controlled oxidation, enhancing H_2_O_2_ selectivity to over 80%.^31^ Adding a hydrophobic polymer layer further improves selectivity by allowing H atoms to pass while blocking molecular H_2_, thus favoring H_2_O_2_ formation on the catalyst surface.^55^ The mechanism of H_2_O_2_ formation on Pd surfaces and clusters has been studied experimentally and theoretically.^56,57^

An advanced “catalytic diffuser” membrane can safely supply reactants and overcome the hydrogen mass transfer limitations seen in dense Pd alloys.^54^ This structure, with graded pore sizes and a fine-porous catalyst layer, boosts H_2_O_2_ productivity to 16.8 mol/h·m^2^ in MeOH and selectivity to 80–90%. An electrically driven membrane–catalyst system sources H_2_ directly from H_2_O (Figure 2).^8^ A Pd-foil membrane separates the electrolysis and hydrogenation chambers, producing H atoms that react with O_2_ to form H_2_O_2_. Optimizing the MeOH/H_2_O ratio and catalyst design increased the H_2_O_2_ concentration 8-fold (from 56.5 to 443 mg/L), with Au–Pd catalysts reducing decomposition. Membrane-based synthesis allows for complete H_2_ consumption, with H_2_ transport through the membrane as the rate-limiting step. While this approach mitigates explosive mixtures, this technology remains far from industrialization.

**Figure 2:**
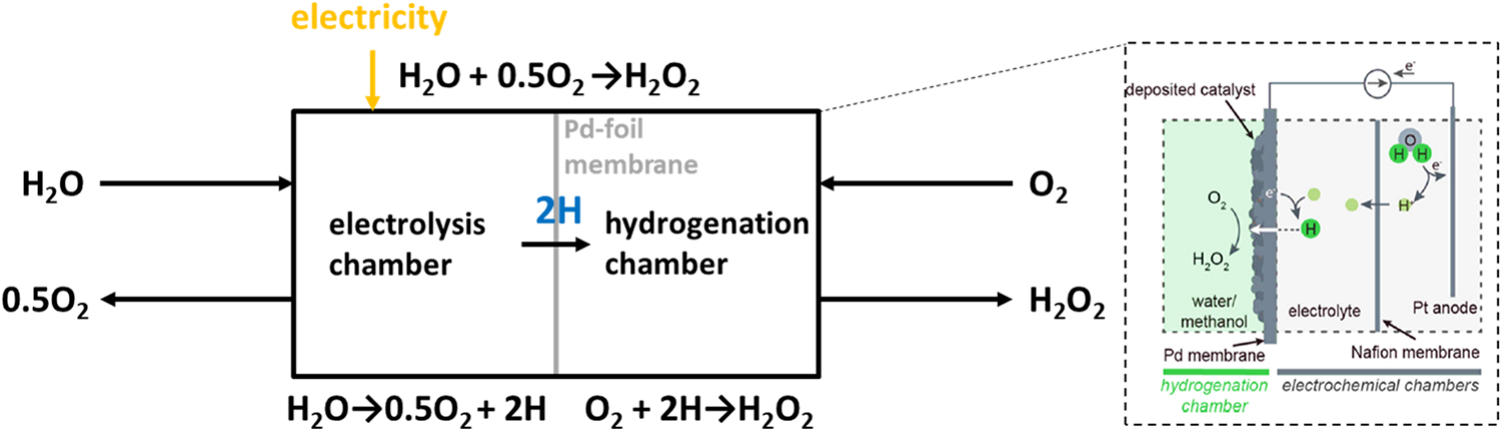
H_2_O_2_ synthesis using a Pd membrane reactor. H_2_ is generated from water and electricity and is separated from O_2_ for hydrogenation via a Pd membrane. Adapted and reprinted with permission.^8^ Copyright 2022 American Chemical Society.

H_2_O_2_ production can be also attained from CO, O_2_, and H_2_O mixtures according to eq 5.^58^

5 $$\mathrm{CO}+O_{2}+H_{2}O\to {\mathrm{CO}}_{2}+H_{2}O_{2}$$

This reaction is thermodynamically favorable (Δ*G*° = −134.2 kJ/mol) and presents an option when a source of CO is readily available.^6^ The feasibility of this concept was first presented using homogeneous palladium triphenyl phosphine complexes as catalysts; however, these showed no potential for an upscaled process. Catalyst deactivation occurred rapidly due to the oxidation of phosphine ligands, resulting in the formation of colloidal palladium nanoparticles in the liquid phase.^59,60^ Au/TiO_2_ can heterogeneously catalyze this reaction, with yields up to 9000 mmol_H2O2_/g_Au_·h. The mechanism and reaction kinetics of the reaction with this catalyst have been investigated.^61^

The advantages, disadvantages, and key product properties for the different thermochemical routes are summarized in Table 2.

**Table 2:** Comparison of Thermochemical Synthesis Methods for H_2_O_2_ Production

**advantages**	**disadvantages**	**H**_**2**_ **O**_**2**_ **productivity and concentration or selectivity**
DSHP		
• high efficiency and selectivity with noble metal catalysts like Pd and Au	• high cost of noble metal catalysts	• productivity: up to 426.7 mmol/g_cat_·h (e.g., Au–Pd catalysts)^46^
• potential for high H_2_O_2_ production rates	• catalyst deactivation due to dissolution in acidic environments	• concentration: up to 10 wt %^17^
• versatility in catalyst and support modifications to enhance performance	• safety concerns with highly concentrated acidic solutions	
	• potential for secondary reactions reducing H_2_O_2_ yields	
**catalytic membranes**		
• high efficiency and selectivity with noble metal catalysts like Pd and Au	• the reaction rate is largely dictated by mass transport limitations	• productivity: up to 16.8 mol/h·m^2^ assuming 70 m^2^/g surface area^54^
• potential for high H_2_O_2_ selectivity	• challenges in achieving high H_2_O_2_ concentrations in the liquid phase	• selectivity: 80–90% with Pd/Ag alloy membrane and hydrophobic polymer layer^54^
• facilitates complete consumption of H_2_, reducing explosive risks	• need for further optimization for industrial scalability	
• innovative designs can potentially improve productivity and selectivity		
**synthesis from CO/O**_**2**_ **/H**_**2**_ **O**		
• utilizes cost-effective and widely available CO as feedstock	• homogeneous catalysts face rapid deactivation and limited turnover numbers, preventing industrial use	• productivity: up to 74.6 mmol/g_cat_·h with Au/TiO_2_ ^61^
• potential for lower energy usage	• limited research on heterogeneous catalysts with issues in selectivity	• concentration: achieved up to 32 wt % with optimized conditions^61^
• homogeneous catalysts offer good selectivity with proper ligand choices	• deactivation of catalysts by water	
• noble-metal-free heterogeneous catalysts also show promising activity	• mass transfer limitations affect overall efficiency	

### Electrochemical

The electrochemical production of H_2_O_2_ proceeds primarily via the two-electron oxygen reduction pathway (2e^–^ ORR, eqs 6 and 7). However, this route is challenging because of the competitive reaction of O_2_ to H_2_O or OH^–^ through the four-electron pathway (4e^–^ ORR, eqs 8 and 9). For both pathways, the use of both acidic and alkaline media is well described in the literature.^62–64^ H_2_O_2_ can also be produced via the two-electron water oxidation reaction (2e^–^ WOR, eqs 10 and 11) at the anode.^62,65,66^ Due to the absence of gaseous reagents, the 2e^–^ WOR requires a simpler experimental setup than the 2e^–^ ORR. However, the 2e^–^ WOR requires a high overpotential (1.76 *V* vs RHE), and it also has a competitive four-electron oxidation (4e^–^ WOR) pathway (eq 12).

6 $$\begin{array}{ll} O_{2}+2H^{+}+2e^{-}\to H_{2}O_{2} & E^{0}=0.68\;V_{\mathrm{RHE}} \end{array}$$

7 $$\begin{array}{ll} O_{2}+2H_{2}O+2e^{-}\to {{\mathrm{HO}}_{2}}^{-}+{\mathrm{OH}}^{-} & E^{0}=0.06\;V_{\mathrm{RHE}} \end{array}$$

8 $$\begin{array}{ll} O_{2}+4H^{+}+4e^{-}\to H_{2}O & E^{0}=1.23\;V_{\mathrm{RHE}} \end{array}$$

9 $$\begin{array}{ll} O_{2}+2H_{2}O+4e^{-}\to 4{\mathrm{OH}}^{-} & E^{0}=0.4\;V_{\mathrm{RHE}} \end{array}$$

10 $$\begin{array}{ll} 2H_{2}O\to H_{2}O_{2}+2H^{+}+2e^{-} & E^{0}=1.765\;V_{\mathrm{RHE}} \end{array}$$

11 $$\begin{array}{ll} H_{2}O\to {\mathrm{OH}}^{•}+H^{+}+e^{-} & E^{0}=2.38\;V_{\mathrm{RHE}} \end{array}$$

12 $$\begin{array}{ll} 2H_{2}O\to O_{2}+4H^{+}+4e^{-} & E^{0}=1.23\;V_{\mathrm{RHE}} \end{array}$$

The electrocatalyst is a vital component for the production of H_2_O_2_ via 2e^–^ ORR and 2e^–^ WOR. Electrocatalysts should have high activity, operating at low overpotential with high current density, and high selectivity, maximizing the 2e^–^ pathways while inhibiting the 4e^–^ pathways. Furthermore, they should display high stability, enabling long-lasting performance and long-term durability at low cost, for the feasibility of industrial-scale applications.^62,67^

### Cathode Materials

To date, numerous catalysts consisting of noble metals, carbon, or their corresponding composites have been developed for the 2e^–^ ORR pathway. Single atom catalysts (SACs) have emerged as a next-generation option. Commonly used cathode materials for H_2_O_2_ production via the 2e^–^ ORR are summarized in Table 3. Generally, noble metal-based catalysts possess excellent activity and selectivity, but the applied metals are costly and scarce.^68,69^ Carbon-based catalysts provide numerous advantages because of their high activity and stability, but structure–performance relationships are challenging due to the complex carbon-based catalytic pathway. Efforts are needed to regulate the structure and functional groups to promote the 2e^–^ ORR.^70–74^ SACs maximize atom utilization and minimize size effects, which strongly enhance electrochemical H_2_O_2_ production, but fundamental knowledge on the reaction mechanism is still insufficient.^70,75–78^

**Table 3:** Summary of Cathode and Anode Materials for H_2_O_2_ Production via 2e^–^ ORR

**materials**		**electrolyte**	***E* versus RHE (*V*)**	***J* (mA/cm** ^**2**^ **)**	**FE** ^a^ **(%)**	**H**_**2**_ **O**_**2**_ **productivity (mg/L·h)**	**refs**
**cathode**							
noble metals	Pt–Hg	0.1 M HClO_4_	0.6	3.0	90		^65^
	Pd/TiO_2_	0.1 M KOH	0.88		86	594	^63^
carbon	MCHS^b^	0.1 M PBS^c^	0.62	1.5	99		^64^
	N-FLG^d^	0.1 M KOH	0.8	15.4	95	936	^66^
	O–CNT^e^	0.1 M KOH	0.72	20	90	3950	^79^
	GF^f^	0.05 M Na_2_SO_4_		12	63	160	^80^
SACs	Co–N–C	0.5 M H_2_SO_4_	0.1	2.9	80		^67^
	Fe–C–O	0.1 M KOH	0.8	0.1	95	∼500	^81^
	Mo-OSG^g^	0.1 M KOH	∼0.7	0.1	95		^82^
**anode**							
metal oxide	BiVO_4_	1 M KHCO_3_	3		35		^83^
	Gd-BiVO_4_	2 M KHCO_3_	3.1	5.2	78	5406	^84^
	CaSnO_3_–CF^h^	2 M KHCO_3_	2.9		90		^73^
carbon	CNFs^i^/NF^j^	1 M Na_2_CO_3_	2.9	72.6	41	165.9	^72^
	BDD^k^/Ti	1 M KHCO_3_	3.5	88.2	28	11,895	^76^
	BDD/Nb	1 M KHCO_3_	3.0	300	87	1,55,856	^77^

a Faradaic efficiency.

b Mesoporous carbon hollow sphere.

c Phosphate-buffered saline.

d Few-layered graphene.

e Carbon nanotubes.

f Graphite felt.

g O/S dual coordinated graphene.

h Carbon felt.

i Carbon nanofiber.

j Nanofiber.

k Boron-doped diamond.

### Anode Materials

Compared with the 2e^–^ ORR pathway, the 2e^–^ WOR requires no O_2_ or aeration and is therefore simpler with greater prospects for practical applications. However, suitable anode catalysts that improve the selectivity of the 2e^–^ WOR and inhibit the competitive 4e^–^ WOR are required. Metal oxides and carbon-based materials have been used as catalysts (Table 3). Generally, stability is the crucial metric due to the high oxidizing potential required for the 2e^–^ WOR. Many metal oxides, like BiVO_4_ and CaSnO_3_, show good results regarding overpotential and H_2_O_2_ production rate. However, high efficiencies are usually based on low current densities.^80,85–89^ Carbon materials demonstrate good H_2_O_2_ activity and selectivity.^90,91^ The stability can be improved with composite carbon materials or diamond-like carbons.^92–95^ The effects of heteroatom dopants and the electrolytes on the catalytic mechanism have been studied.^96,97^

### Cell Configurations

Apart from efficient anode and cathode catalysts, the cell configuration is critical for effective electrosynthesis of H_2_O_2_, particularly in industrial-scale applications.^98^ Extensive efforts have been made to design effective cell devices. A continuous flow of electrolyte to prevent accumulation and decomposition of in situ-generated H_2_O_2_ is essential. Additionally, the cell setup should enhance mass transfer rates for an efficient 2e^–^ ORR. Different cell configurations for H_2_O_2_ production have been investigated (Figure 3). A review of the performance of the different cell configurations is available.^99^

**Figure 3:**
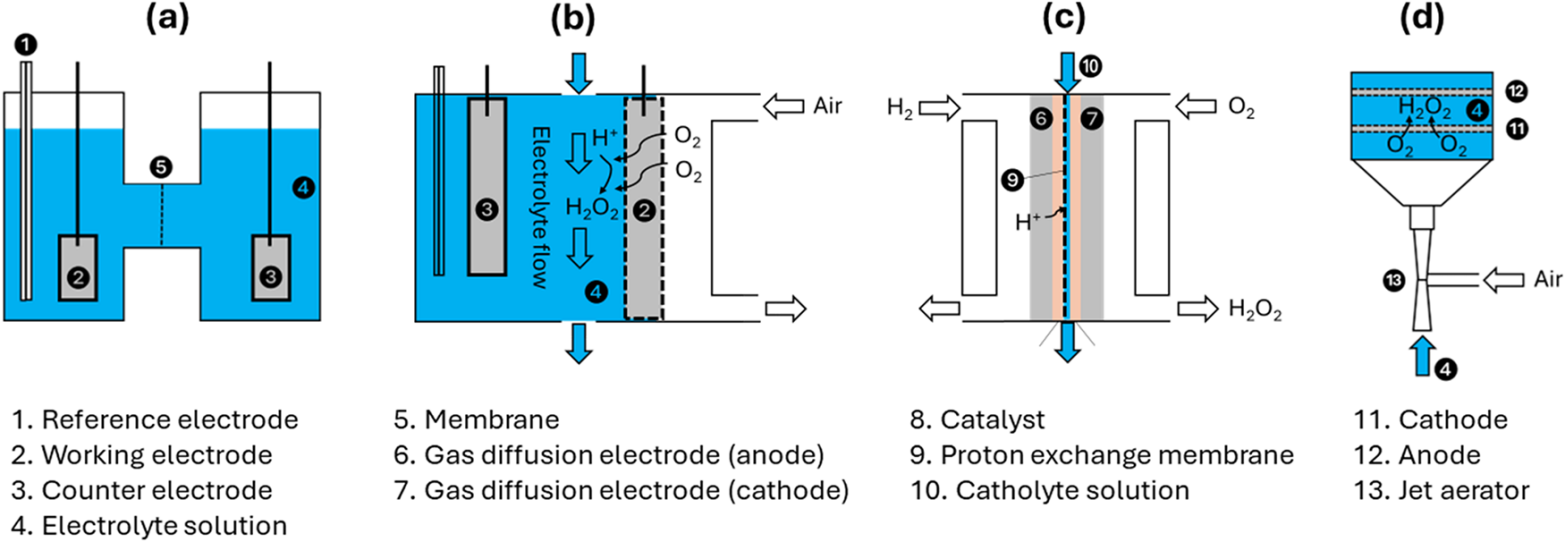
Illustrations of the different configurations of electrochemical cells for H_2_O_2_ production. The blue color represents liquid-phase flows, while the colorless areas represent gas-phase flows. (a) H-cell, adapted with permission.^76^ Copyright 2020 American Chemical Society; (b) gas–liquid separated flow cell, reproduced^100^ with permission from the Royal Society of Chemistry; (c) dual-chamber reactor with membrane, reproduced^89^ with permission from Springer Nature BV; and (d) dual-chamber reactor with venturi-based jet aerator, reproduced^94^ with permission from Elsevier Science and Technology Journals.

The H-type cell (Figure 3a) is commonly used in the laboratory. A proton-exchange membrane (PEM) is incorporated to separate the anode and cathode, which makes the synthesis of pure H_2_O_2_ solution feasible. However, the small, fixed volume and insufficient oxygen mass transfer limit the production of H_2_O_2_ at practical rates. Additionally, the low mass transfer and accumulation of H_2_O_2_ near the electrode surface lead to H_2_O_2_ decomposition.^80^

Alternative flow cell configurations have been developed,^66,83^ e.g., a gas–liquid separated flow cell (Figure 3b). Due to the absence of gas transfer in the liquid, O_2_ can pass through the electrode rapidly and immediately be reduced at the active sites. Additionally, the circulating liquid favors the transfer and collection of the produced H_2_O_2_, thus avoiding its decomposition at the electrode while achieving a high concentration.^101^ Similarly, a gas diffusion electrode (GDE) or natural air diffusion electrode (NADE), which contains a hydrophobic gas diffusion layer and acts as a membrane between O_2_ and liquid electrolyte, achieves rapid O_2_ transport to the active sites and enhances the stability of the electrodes by mitigating H_2_O_2_ corrosion of the catalyst.^102,103^ Compared to the normal GDE, a superhydrophobic NADE enables O_2_/air to naturally diffuse to the electrode interface and shows longer operation stability.^104,105^

In a dual-chamber reactor with the cathode and anode chambers separated by a PEM (Figure 3c), the ion diffusion path and solution resistance are reduced by a narrow-gap cell based on a membrane electrode assembly (MEA). The liquid electrolyte is introduced into a narrow channel between the electrode and the membrane.^99^ An alternative solution is a flow-through device that enhances the H_2_O_2_ desorption from the active sites, reducing the possibility of H_2_O_2_ degradation.^99^ The addition of a venturi-based jet aerator (Figure 3d) can supply extra O_2_ without additional energy consumption.^94^

A solid-state electrolyte (SSE) device is much like a dual-chamber reactor (Figure 3c) and contains a functionalized proton-conducting copolymer microsphere in the middle chamber, separated from the electrodes by an anion and a cation exchange membrane (AEM and CEM, respectively). Produced ions are driven toward the SSE layer by Coulombic attraction and the internal electric field and recombine rapidly to form H_2_O_2_. Flushing the SSE with pure H_2_O can provide H_2_O_2_ solutions without ionic impurities.^106^ Based on the above cell structure, a wide range of H_2_O_2_ concentrations from 0.3 to 20 wt % were obtained while maintaining activity and selectivity in the SSE for 100 h.^95^ By adding a boron-doped carbon cathode catalyst, the more economical 2e^–^ ORR was favored over the 4e^–^ ORR.^106^ However, large-scale implementation remains difficult due to the internal resistance and permeability challenges.^72^

To date, the development of electrochemical reactor system designs is less advanced than the catalyst design. H-cells are useful for catalyst screening, while flow cells are more promising for high-throughput H_2_O_2_ production and improve selectivity by carrying H_2_O_2_ away from the cathode surface before further reduction occurs. The reactor design should also consider the energy cost for the circulation of O_2_, which can be assisted with the use of GDEs. Additionally, the MEA design tends to have a high single-pass efficiency due to a decreased diffusion path. While SSE designs enable highly pure H_2_O_2_ products with low concentrations, there are still permeability issues to be solved.

### Bioelectrochemical

Bioelectrochemical systems (BESs), which integrate microbial metabolism with electrochemistry, are considered a promising approach for obtaining energy from biomass. Two main types of BESs are microbial fuel cells (MFCs) and microbial electrolysis cells (MECs). The common principle is that electroactive bacteria initiate electrochemical reactions from the organic substrate in the anodic chamber. The electrons are transferred to the cathodic chamber, which is used to produce bioelectricity in MFCs or H_2_ in MECs, see Figure 4. MECs require exogenous energy to overcome thermodynamic barriers.^107,108^ When O_2_ is utilized as the electron acceptor at the cathode, H_2_O_2_ is produced via the 2e^–^ ORR.^109^ By adjusting working modes, both MFCs and MECs have been applied for H_2_O_2_ synthesis via the 2e^–^ ORR.^108,110^

**Figure 4:**
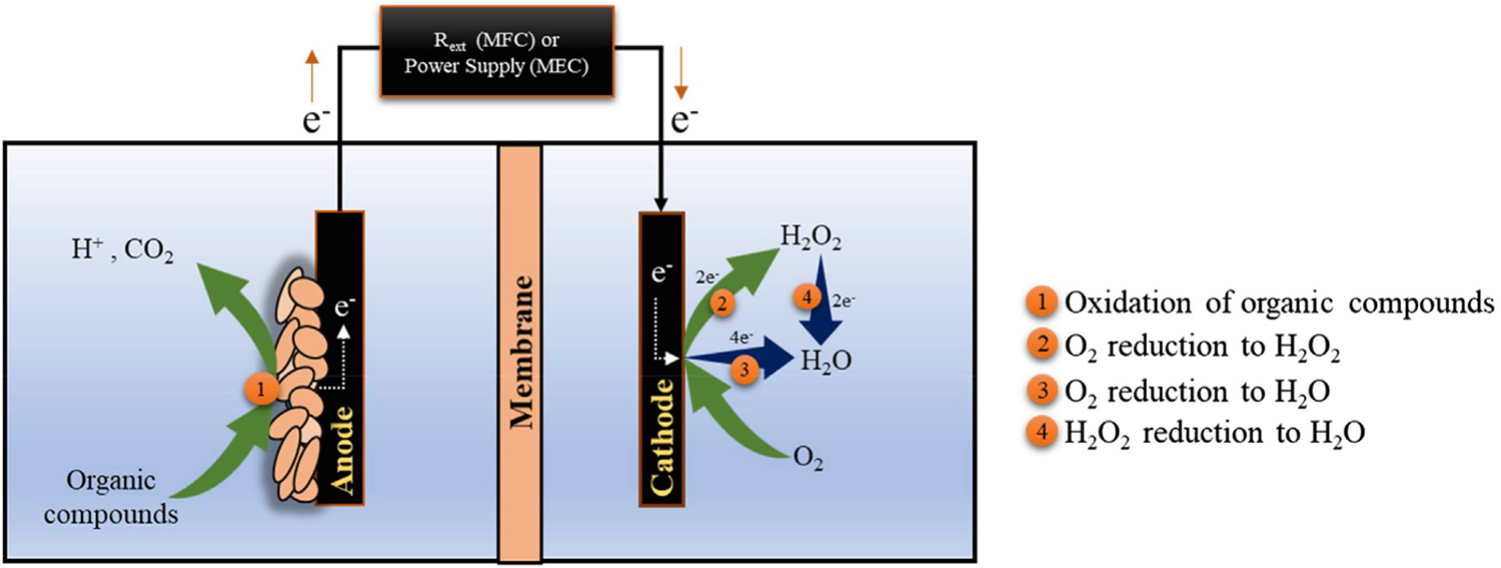
Mechanistic representation of H_2_O_2_ production via BESs. Reprinted^111^ with permission from Elsevier.

The anodic electrode surface should enable the growth of electroactive bacteria and allow a high electron transfer rate. Thus, certain characteristics, such as biocompatibility, appropriate mechanical strength, good conductivity, chemical stability, and large surface area, are important for the selection of anode materials.^112^ The most commonly used material that satisfies the above requirements is carbon, including carbon cloth, carbon felt, carbon fiber, graphite felt, and graphite fiber.^112,113^ To strengthen bacteria attachment on the anode, modifications of the electrode surface by NH_3_ pretreatment and nitrogen doping have been investigated, which enhance the microbial interaction through hydrogen bonds or van der Waals forces.^114–116^ In addition, nanomaterials such as carbon nanotubes (CNTs) have proven to enhance the bacterial attachment to the electrodes.^117^ In general, the electrode materials used in MFCs and MECs possess similar properties. CNT and graphene are commonly used in the anodic materials of MFCs, while graphene-based derivatives and composites are mostly reported in MECs.^107^

Both pure organic and waste organic substrates can be used as anolytes in H_2_O_2_-producing BESs. Substrates such as acetate, glucose, and cellulose are commonly used as a carbon source for the microorganisms. An H_2_O_2_ production rate of 3.3 mg/L·h was achieved in an MEC with the use of municipal wastewater.^118^ However, primary sludge produces more H_2_O_2_ due to its higher chemical oxygen demand content and an increase in electrons transferred for H_2_O_2_ generation.^118^ Nonetheless, the highest production rate was achieved with a pure substrate. The addition of acetate resulted in a H_2_O_2_ production rate 40 times higher than that for real wastewater,^119^ which could be attributed to the high biodegradability of acetate or to the low conductivity and high internal resistance of wastewater.^112^

Like anode materials, carbon-based materials are also preferred for cathodes.^113^ It was found that graphite particle electrodes exhibited the optimum performance for H_2_O_2_ synthesis,^120^ since graphite electrodes inhibit the 4e^–^ ORR and prevent further H_2_O_2_ decomposition to H_2_O.^108^ An extra coating layer, such as a nonprecious metal, often enhances H_2_O_2_ production.^113^ Studies indicated a 10-fold higher H_2_O_2_ production rate using a GDE over a graphite cathode electrode.^119^ Additionally, several air-cathodes, such as a graphene-based air-cathode^121^ and a pure carbon black air-cathode,^122^ showed promising results in H_2_O_2_ production.

The properties of the catholyte also substantially influence the production and stability of H_2_O_2_.^108^ The stability of H_2_O_2_ is quite sensitive to the catholyte pH, where a neutral pH is favorable for the microorganism metabolism and lowers the overpotential (pH gradient) between the two chambers, even though most studies report the highest H_2_O_2_ concentration in an acidic environment.^68^ As an alternative, tap water was utilized as a catholyte for H_2_O_2_ production to avoid secondary pollution and the high cost of electrolytes.^108^

BESs generally use the dual-chamber system separated with an ion exchange membrane, preventing the exposure of microorganisms to H_2_O_2_.^111^ Table 4 lists examples of BESs for H_2_O_2_ production. The stability of H_2_O_2_ could be maintained when CEMs were used instead of AEMs,^123^ due to the transport of protons from the anode to the cathode chamber to lower the cathodic pH. However, AEMs show unique advantages with real wastewater,^118,119^ as it avoids the diffusion of unwanted cations (e.g., Ca^2+^, Mg^2+^) and the acceleration of H_2_O_2_ decay by metal catalysis.^113,114^

**Table 4:** Summary of Different Types of BESs for H_2_O_2_ Synthesis

					**H**_**2**_ **O**_**2**_ **production**		
**system**	**anode/cathode**	**substrate/catholyte salt**	**reactor volume (anodic/cathodic)**	**membrane**	**rate (mg/L·h)**	**conc.** ^a^ **(mg/L)**	**refs**
MFC	carbon felt/graphite	acetate/Na_2_SO_4_	32 mL/50 mL	CEM	1.5	37.3	^123^
MFC	graphite/graphite	acetate Na_2_SO_4_	125 mL/125 mL	CEM	0.25	2.1	^124^
MFC	graphite/graphite	glucose/Na_2_SO_4_	80 mL/80 mL	CEM	6.6	80	^125^
MEC	carbon cloth/GDE	acetate/NaCl	200 mL/18 mL	AEM	4.2	3800	^118^
MEC	graphite/GDE	domestic ww^b^/NaCl	9.4 mL/9.4 mL	CEM	3.2		^126^
MEC	carbon felt/graphite	acetate/Na_2_SO_4_	32 mL/50 mL	CEM	88.2	705.6	^127^
MEC	carbon fiber/GDE	acetate/tap water	100 L/10 L	AEM	0.02	9	^113^
MEC	graphite/graphite	acetate/Na_2_SO_4_	10 L/10 L	CEM	10.8	420	^128^

a Concentration.

b Wastewater.

### Plasmatic

Unlike most other processes that employ catalysts, plasma-assisted processes generate H_2_O_2_ by igniting plasma with electrical energy. This partially ionized gas forms H_2_O_2_ under ambient conditions without complex catalysts or solvents, providing a cleaner production method. H_2_O_2_ can be formed either through the direct plasma activation of H_2_ and O_2_ in the gas phase (eq 13) or by a plasma/H_2_O interaction (eq 14).^129^

13 $$\begin{array}{ll} H_{2}+O_{2}\to H_{2}O_{2} & \Delta H=1.95\;\mathrm{eV}/\mathrm{molecule}\;\mathrm{or}\;1.6\;\mathrm{kWh}/\mathrm{kg} \end{array}$$

14 $$\begin{array}{ll} 2H_{2}O\to H_{2}+H_{2}O_{2} & \Delta H=3.2\;\mathrm{eV}/\mathrm{molecule}\;\mathrm{or}\;2.5\;\mathrm{kWh}/\mathrm{kg} \end{array}$$

Activation of H_2_/O_2_ in a dielectric barrier discharge (DBD) is generating interest since it promotes H_2_O_2_ formation at atmospheric pressure.^130^ A proposed reaction mechanism for H_2_O_2_ formation in a double DBD reactor^131^ is shown in eqs 15–19.

15 $$H+O_{2}\to {\mathrm{HO}}_{2}$$

16 $$2{\mathrm{HO}}_{2}\to H_{2}O_{2}+O_{2}$$

17 $$O+H_{2}{\;}^{*}\to \mathrm{OH}+H$$

18 $$H+O_{2}{\;}^{*}\to \mathrm{OH}+O$$

19 $$\mathrm{OH}+H_{2}\to H+H_{2}O$$

HO_2_ is the main intermediate species in H_2_O_2_ formation (eqs 15 and 16). Concurrently, H_2_O is also formed via a second pathway (eqs 17–19). The synthesis of H_2_O_2_ is mainly driven by the composition of the gas mixture, the reactor geometry, and the discharge characteristics. Low H_2_O_2_ selectivity, relatively high energy demand, and safety issues are the main bottlenecks of H_2_/O_2_ gas-phase plasma activation.

Direct H_2_O_2_ synthesis in a nonequilibrium plasma environment has been studied using an atmospheric pressure DBD reactor.^132^ This system features a high-voltage (HV) copper electrode covered with a Pyrex tube and a 1.4 wt % NaCl aqueous solution as a liquid grounding electrode, which also serves as a coolant. The grounding electrode prevents thermal H_2_O_2_ decomposition, while the Pyrex tube avoids reactions between O_2_, H_2_O_2_, and the metal electrode, improving yield and selectivity.^132^ Using a H_2_/O_2_ mixture with 3 mol % O_2_ (below the 6% explosive limit of H_2_/O_2_ mixtures, *vida infra*), H_2_O_2_ selectivity increases from 3.5 to 56.3%. However, despite the selectivity enhancement, the energy efficiency is poor due to significant heat loss (80 kWh/kg_H2O2_ versus the theoretical energy consumption of 1.6 kWh/kg_H2O2_), and the H_2_O_2_ productivity is restricted by the low O_2_ content. By replacing the metal HV electrode in the DBD reactor with a saturated NaCl solution covered by a thin Pyrex tube (Figure S1), high-purity H_2_O_2_ can be safely produced with 64% selectivity and 62 wt % concentration (37 g_H2O2_/L·h) using 14.3 mol % O_2_ content and 19 kWh/kg_H2O2_ energy demand.^71,131^

H_2_O_2_ selectivity can also be controlled by adjusting the specific energy input (SEI), with a lower SEI favoring H_2_O_2_ selectivity. However, low SEI leads to low O_2_ conversions and H_2_O_2_ yields featuring high energy demand.^133^ A potential solution may be the injection of Ar and H_2_O into the plasma, serving as an effective energy carrier and enhancing the plasma chemistry, respectively. Up to 3.5 times higher O_2_ conversion (60%) and H_2_O_2_ yield (53.4%) can be attained by applying 12.8 kWh/kg_H2O2_.^134^

Discharge inside or in contact with the liquid phase triggers reactions at the plasma–liquid interface, generating reactive species in both the gas and bulk liquid phases and enabling electrochemical phenomena at the interface. Plasma–liquid interactions influenced by the discharge type are shown in Figure 5.^135^

**Figure 5:**
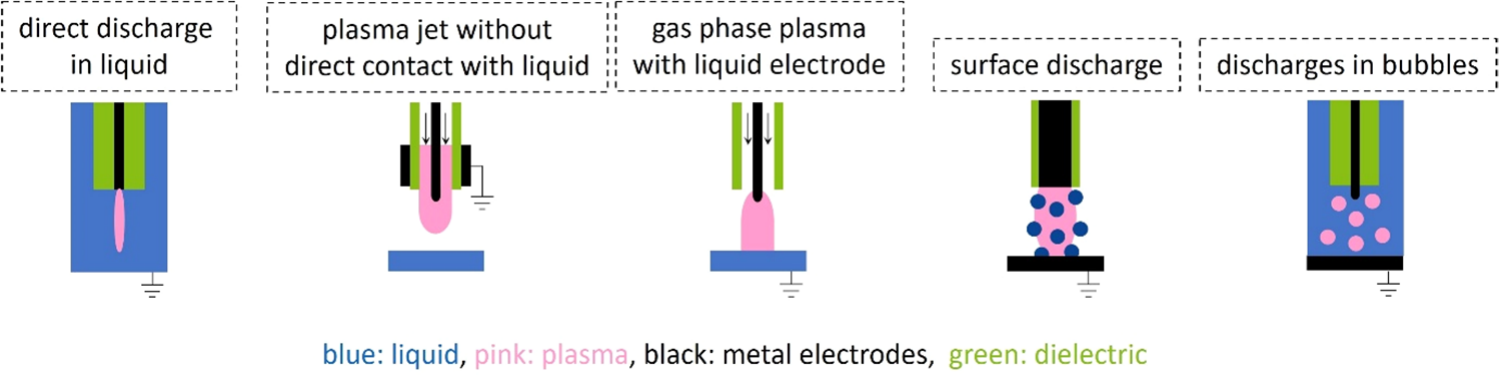
Schematic of different discharges used in plasma–liquid interactions, redrawn.^135^ ©IOP Publishing. Reproduced with permission. All rights reserved.

In plasma–liquid (aqueous solution) systems, H atoms and hydroxyl radicals are generated via H_2_O dissociation. The H_2_O_2_ formation rate is driven by the H_2_O concentration in the plasma phase and increases almost linearly with the amount of applied power,^129^ as evaporation, sputtering, and the electric field are enhanced (Figure S2). It is not clear, however, which factor is dominant. Details on H_2_O_2_ formation pathways in plasma-liquid/aqueous solutions can be found elsewhere.^135–138^

The estimated energy demand for H_2_O_2_ formation can range between 125 and 5000 kWh/kg_H2O2_ depending on the discharge type,^129^ but recent results have improved that, e.g., 116.3–212.8 kWh/kg_H2O2_.^139,140^ The H_2_O_2_ concentration is affected by the pH of the liquid^139,141^ and increases linearly with the total energy input.^140^ By varying those parameters, the energy yield can be maximized, but erosion phenomena on the metal electrodes may occur via electrochemical reactions (anodic oxidation followed by electrolyte dissolution) or thermal effects.^140,142–144^

In gas-phase discharges above aqueous solutions, both the H_2_O_2_ production rate and energy yield increase with higher discharge current and liquid conductivity, which can be adjusted using chemicals like NaCl or NaOH.^145^ The H_2_O_2_ concentration, production rate, and energy yield (energy efficiency) are enhanced by longer residence times, higher power, larger gas–liquid interface areas, and higher reactor temperatures, which promote H_2_O evaporation (up to the decomposition point of H_2_O_2_).^9^ Inert gases also influence H_2_O_2_ production by expanding the plasma interface film region at the plasma–liquid boundary and reducing H_2_O_2_ degradation due to a lower specific energy density.^146^ DBDs are the common plasma reactors used, while recently a modular, adaptable and continuous DBD plasma tubular microreactor was developed providing advantages like increased heat and mass transfer rates, improved reaction control, and limited waste generation.^9^ Indicatively, H_2_O_2_ energy demand and concentrations recently achieved ranges between 143 and 588 kWh/kg_H2O2_ and 0.4 and 27 mM, respectively, by applying 0.9–5 W.^9,145–147^ The main disadvantages of gas-phase discharges over a liquid are the erosion of the electrodes associated with direct liquid-phase electric discharge^140^ and the low mass transfer and energy yield, making the upscaling for industrial application difficult.^129^

Plasma discharge in bubbles enhances yields and reduces electrode erosion^129^ by improving gas-to-liquid mass transfer through larger interfacial areas, longer residence times, and higher internal pressures.^129,137^ Microsized O_2_ or Ar bubbles are preferred for better mass transfer.^137,148^ This method generates large plasma volumes in water with lower voltage and energy use.^148,149^ The H_2_O_2_ formation rate is proportional to the input power.^149^ Recently, a 164.6 mg_H2O2_/h production rate was achieved, with 111 kWh/kg_H2O2_ energy demand, using an eight-microhole reactor powered by solar panels, connected with a secondary reactor equipped with a 2D-TiO_2_/g-C_3_N_4_ photocatalyst.^150^ Table 5 summarizes the different plasma-assisted synthesis reactors and their SEI.

**Table 5:** Comparison of Specific Energy Input (SEI) for H_2_O_2_ Production Using Different Plasma Reactors

**reactor type**	**feed**	**SEI (kWh/kg**_**H**_**2**_ **O**_**2**__ **)**	**refs**
DBD with metal HV electrodes	gas-phase H_2_/O_2_	80	^132^
DBD with liquid (aqueous solution) electrodes	gas-phase H_2_/O_2_	19	^131^
DBD with metal HV electrodes	gas-phase H_2_/O_2_/Ar/H_2_O	12.8	^134^
pin to pin (underwater plasma discharge); pin hole	liquid H_2_O; phosphate-based liquid electrolyte	116.3–212.8	^139,140^
Eight-microhole reactor (underwater plasma bubbles)	liquid H_2_O with Ar bubbles	111	^150^

## Holistic Process Benchmarking

### Energy Footprint

Energy consumption is one of the indicators to comprehensively understand the feasibility of different processes, and the corresponding required energy inputs for both the AO process and emerging technologies are presented in Table 6. It has been reported that 0.8–1.6 kWh/kg_H2O2_ (2.9–5.7 GJ/1000 kg_H2O2_) at 50% product concentration is required for the AO process^151^ after energy optimizations, which saved up to 1.1 kWh/kg_H2O2_. The biggest energy contributors are purification (∼0.4 kWh/kg_H2O2_) and gas compression (∼0.4 kWh/kg_H2O2_). However, these numbers exclude the production of H_2_. If H_2_ is produced by SMR, the total energy consumption becomes 1.3–2.1 kWh/kg_H2O2_ and, for electrochemically produced H_2_, 3.8–5.5 kWh/kg_H2O2_.^152^

**Table 6:** Comparison of Energy Consumption between Different H_2_O_2_ Production Processes

**process**		**energy input (kWh/kg_H2O2_)**	**scale**	**refs**
AO process (SMR-H_2_)		1.3–2.1	state-of-the-art, industrial scale	^155,156^
AO Process (e-H_2_)^a^		3.8–5.5		^152^
emerging technologies	plasma	12.8–1429	lab scale	^134,157^
	electrochemical	3.1–10^b^	lab scale	^99,154^
	MECs	0.3–56^b^	lab scale, pilot scale	^111,128^

a Electrochemically produced.

b Electricity consumption from an electrochemical cell.

The reported energy consumption data for electrochemically produced H_2_O_2_ varies significantly, e.g., from 3.1–60 kWh/kg_H2O2_. This depends on many aspects, such as the applied electrode materials, cell configurations, and operating conditions. For instance, an energy input of 4.6 kWh/kg_H2O2_ at 20 mA/cm^2^ was reported, compared with 19.4 kWh/kg_H2O2_ at 240 mA/cm^2^, which is a direct influence of the decrease in the current efficiency.^153^ An average energy consumption of 9.3 kWh/kg_H2O2_ was reported for a 46-day test.^154^ Therefore, when operating more efficiently, the energy consumption is more likely to range from 3.1–10 kWh/kg_H2O2_. For BESs, an external potential is applied to enhance H_2_O_2_ production via an MEC.^107^ Most studies on MECs are still conducted at lab scale, and maximum energy inputs up to 56 kWh/kg_H2O2_ were found, although for a small-scale 26 cm^2^ prototype and 100 mL solution, an energy input of 0.3 kWh/kg_H2O2_ was reported.^128^

Energy consumption for plasma-assisted H_2_O_2_ formation ranges between 12.8 and 1429 kWh/kg_H2O2_, making it the most energy-intensive emerging process. The energy demand for H_2_O_2_ synthesis through H_2_ and O_2_ plasma activation is lower (12.8–80 kWh/kg_H2O2_) than that for H_2_O activation (111–1429 kWh/kg_H2O2_), as more energy is required to facilitate phenomena such as H_2_O evaporation. Enhancing gas–liquid mass transfer and extending the residence time can significantly improve the energy efficiency of plasma-based processes.

However, for various emerging technologies, the energy input values shown in Table 6 were only calculated from the voltage input of the electrochemical cell and did not account for the energy requirements of other equipment such as pumps, heaters, cooling equipment, and concentration and purification units. Additionally, for the AO process, the best state-of-the-art techniques are used and emerging technologies still need to be further optimized, which could lead to a reduced energy input.

### Techno-Economic Feasibility

Although various emerging technologies such as electrochemical, BES and plasma processes are potentially interesting alternatives for industrial H_2_O_2_ production, most reports mainly cover small-scale studies with very low H_2_O_2_ concentrations. Only limited information was found on economic assessments of capital and operating costs of emerging technology at an industrial scale. For some technologies (BESs, plasma), the technology readiness level is still too low and energy consumption too high for a meaningful or positive economic assessment. As one of the most representative emerging technologies, electrochemical processes exhibit excellent performance under certain conditions. Based on the results of H_2_O_2_ electrosynthesis obtained at lab scale, the techno-economic feasibility of industrial implementation has been evaluated by some researchers for different scenarios (Table 7).

**Table 7:** H_2_O_2_ Production Cost Estimates for Emerging Technologies versus State-of-the-Art

**type of process**		**GDE** ^154^	**GDE** ^150^	**2e- ORR** ^152^	**2e- ORR (reworked)** ^152^	**AO process** ^158^	**AO process** ^156^
capital cost (EUR/ton)	electrolyzer	276	n.a.	121	1103	276	n.a.
	other units^a^	OSBL	OSBL	OSBL			
operational cost (EUR/ton)	raw materials	1214	n.a.	14	811	368	n.a.
	utilities			305	375		
	others			103	2247		
manufacturing cost (EUR/ton)		1490	810	543	4536	644	1104

a OSBL = outside battery limits (not considered).

For instance, a total cost of 1490 EUR/ton (∼1620 USD/ton)[Fn fn1] for H_2_O_2_ production with a GDE at 150 mA/cm^2^ was estimated, which comprises a capital cost of 276 EUR/ton (∼300 USD/ton) and an operating cost of 1214 EUR/ton (∼1320 USD/ton).^159^ In contrast, lower estimated H_2_O_2_ production costs of 810 EUR/ton (∼880 USD/ton) were based on the required replacement of the GDE electrode every 46 d.^154^ However, the production capacity was not mentioned in either scenario. These manufacturing costs were most likely underestimated by neglecting additional costs for heat exchangers, pumps, separation units, etc. A techno-economic analysis of a H_2_O_2_ electrosynthesis in combination with a purification process to produce 70 wt % H_2_O_2_ was performed.^156^ The estimated production cost was 543 EUR/ton (∼590 USD/ton), which is much lower than that of the AO process (1104 EUR/ton, ∼1200 USD/ton). Although the target system boundary comprised separation and concentration units, the outcome is highly dependent on the economic input parameters that were used, such as an assumed electrolyzer life span of 30 years.

For an economic comparison of the reported H_2_O_2_ electrosynthesis process^156^ with the AO process, the available experimental data from the literature and chosen industrial reference input values were recalculated (Table S1). The designed process, which is composed of electrochemical, separation, and concentration units, corresponds to a typical minimum industrial target to produce 35 wt % H_2_O_2_ (25 ton/d on a 100% H_2_O_2_ basis). In the electrochemical cell, a Na_2_SO_4_ solution is used as the circulating electrolyte for both chambers. In the anode chamber, O_2_ is generated via the 4e^–^ WOR, and 75% of the gas is assumed to be recovered in the cathodic reaction. Meanwhile, H_2_O_2_ is produced via the 2e^–^ ORR in the cathode chamber, and the concentration reaches 4.6 wt % under certain conditions. The H_2_O_2_ mixture is then fed into a stripping unit where H_2_O_2_ is separated from Na_2_SO_4_. The remaining Na_2_SO_4_ solution is sent to the evaporator to remove the excess H_2_O before returning to the cathode chamber as the recycled electrolyte. The H_2_O_2_ is purified and concentrated to 35 wt % in a rectification column. All related process parameters are listed in the Supporting Information (Table S2). The recalculation of capital and operational costs was completed based on an industrial standard.^160^

The recalculated manufacturing cost for industrial H_2_O_2_ production via an electrochemical process is greater than eight times that of the reported value. This indicates that both the reported capital and operational costs are significantly underestimated. According to the study,^156^ only the electrolyzer and separation units are considered for the calculation of the capital cost. In contrast, heat exchangers, various buffer and storage vessels, pumps, and other auxiliaries are also included in the recalculations. The recalculated value of the electricity cost is slightly higher than that reported in the literature, even though a lower electricity price was adopted during the recalculation, since only electricity from the electrolyzer is counted in the study, excluding the superheated steam and chillers needed for H_2_O_2_ separation and purification. Additionally, increases in the relatively low benchmark prices for Na_2_SO_4_ (1.8 EUR/ton) and O_2_ (40 EUR/ton) adopted in the reference result in higher recalculated values. Finally, the costs for utilities, labor, and maintenance are considered to be underestimated, which again increases the recalculated values.

Others^161^ confirm the challenge to make emerging technologies for the electrochemical production of H_2_O_2_ economically viable. Here, it was anticipated that the cost of electrochemical H_2_ production could be lowered through a combination with selective H_2_O oxidation to H_2_O_2_ over boron-doped diamond (BDD) electrodes. It was found that despite H_2_O_2_ being a more valuable coproduct than O_2_, the levelized cost of H_2_ was significant (62 USD/kg), mainly because of the high market price of BDD electrodes.

### Environmental Footprint

While H_2_O_2_ is vital for many applications due to its strong oxidation potential and benign degradation products, its production through the AO process poses significant environmental challenges, including high energy consumption, hazardous waste generation, and fossil fuel reliance. While promising alternatives are the emerging electrochemical processes that are ideally fueled by renewable electricity, they are not yet industrialized. Currently, industry focuses on more energy-efficient and cost-effective applicable solutions, which involve transitioning to the consumption of lower carbon footprint H_2_ and to a renewable electricity supply for the main utilities required to produce H_2_O_2_ via the AO process. Examples include green H_2_ production via electrolysis powered by renewable electricity (e.g., photovoltaics) and the production of steam using heat pumps or electrified boiler technologies. Key H_2_O_2_ players, such as Evonik,^162^ Solvay,^163^ and Nouryon^164^ have already invested in such technologies.

Studies focusing on the environmental performance of H_2_O_2_ production are limited; only one life cycle assessment (LCA) study^165^ could be found in an international peer-reviewed journal, highlighting the research gap in the field. Alternatively, the environmental impact of the AO process can be retrieved from LCA databases, like Ecoinvent.^166^ In addition, one can find publicly available environmental product declaration data^167^ carried out for the benefit of H_2_O_2_ production companies. However, in that case, it is difficult to rationalize the data due to the limited detailed information provided.

According to the Ecoinvent database, the calculated global warming potential midpoint (GWP (100)) impact of the AO process is 1.79 kg of CO_2‑equiv_/kg_H2O2_. The data are based on the European market (average data from eight European producers), and the boundary limits extend from the extraction of natural resources to H_2_O_2_ ready to leave the gate of the production site (“cradle-to-gate” approach). The different process contributions are shown in Figure S3. The production process for the required H_2_ (i.e., via SMR) is the main polluting driver, contributing 44% of the climate change impact of the AO process. H_2_ impact is mainly attributed to the direct CO_2_ emissions primarily generated from the combustion of natural gas to meet the energy requirements of the endothermic SMR process and from the CO_2_ formed as a byproduct during the reforming stage.^166^ Grid electricity, mixed-fossil-based steam, and natural gas-based heat are the main utilities used by the AO process, contributing 10.3, 27.8, and 7.8%, respectively, to the overall climate change impact.

The impact of the H_2_ source on the environmental performance of the AO process was studied.^165^ Focusing on H_2_O_2_ production in China, a cradle-to-gate LCA study investigated four different scenarios for H_2_ production: SMR, coal coking and gasification, cracking of fossil-based hydrocarbons, and as a byproduct of the chlor-alkali electrolysis (CAE). To develop a Chinese localized LCA, real life cycle inventory (LCI) data from seven Chinese companies were retrieved, except for information on organic chemical production that was obtained from Ecoinvent. Given that China’s energy sector is mainly based on coal-fired power plants, electricity and steam were considered as sourced from coal. Climate change, water use, human toxicity-non cancer, and fossil resources were defined as the main impact categories, and the normalized results per 1 ton H_2_O_2_ (27.5 wt %) were presented using midpoint and end point indicators.

Using the same four H_2_ sources listed above and adjusting the results^165^ to 1 kg H_2_O_2_ (100%), the GWP (100) impact of H_2_O_2_ varies between 1.61 and 2.50 kg_CO2‑equiv_/kg_H2O2_, depending on the H_2_ source. Figure S4 shows the breakdown of the carbon footprint (expressed as kg_CO2‑equiv_/kg_H2O2_) of these different H_2_ sources on the AO process. Τhe key pollutants were the coal-based production of electricity and steam, contributing 56–86% of the GWP (100) impact, depending on the H_2_ source. The carbon footprint associated with H_2_ production decreases in the order of 14.5 kg_CO2‑equiv_/kg_H2_ (SMR) > 4.3 kg_CO2‑equiv_/kg_H2_ (coal coking and gasification) > 1.9 kg_CO2‑equiv_/kg_H2_ (cracking) > 0.74 kg_CO2‑equiv_/kg_H2_ (CAE). The H_2_ contribution is limited (3%) when green H_2_ (originating from CAE) is used, leading to the lowest overall carbon footprint for the AO process (1.61 kg_CO2‑equiv_/kg_H2O2_).^168–170^ On the other hand, while the highest footprints belong to SMR and coal coking and gasification, application of carbon capture and storage methods reduces the environmental impact of the H_2_ production and consequently improves the environmental performance of H_2_O_2_ production, for example, by 69% for SMR and by 79% for coal gasification.^169^

An alternative, effective strategy to enhance the sustainability of the AO process involves minimizing its dependence on conventional electricity, a key pollution source. This can be achieved by increasing the penetration of renewable energy sources in the production process while still producing H_2_ via SMR (see Figure S5). By switching from lignite-based electricity to 100% renewable electricity (wind-harvested), the overall AO GWP (100) decreases by 30%.

Table 8 summarizes the LCA of the AO process utilizing H_2_ formed via SMR or CAE (differentiated by the energy source and geographical area) for the impact categories of global warming, fossil resource scarcity, and water consumption. The results are based on the above study^165^ and on the Ecoinvent database.^166^ When H_2_ is formed via SMR, the use of coal-based electricity increases the GWP (100) impact of the AO process. However, the effect on fossil resource depletion and water consumption categories is lower than that for grid electricity. The same applies when H_2_ is produced through CAE. Using green H_2_, the AO process has the lowest environmental impact among the different H_2_ production routes discussed (e.g., SMR, coal coking, and gasification), which could be even lower if grid electricity is replaced by 100% renewables (approximately 11% lower in case of wind-harvested electricity).^166^

**Table 8:** Life Cycle Environmental Impact for H_2_O_2_ Production Process (AO) Utilizing H_2_ Formed via SMR or CAE (Differentiated by the Energy Source and Geographical Area)^a^,^b^ ^165,166^

			**impact categories**		
**H**_**2**_ **source**	**electricity and steam source**	**geographical area**	**global warming, kg_CO2‑equiv_/kg_H2O2_**	**Fossil resource scarcity, kg_oil‑equiv_/kg_H2O2_**	**water consumption, m^3^/kg_H2O2_**
SMR	coal coking and gasification	China	2.5	0.455	0.035
SMR	grid electricity and fossil-based steam	Europe	1.79	0.60	0.076
CAE	coal coking and gasification	China	1.61	0.334	0.036
CAE	grid electricity and fossil-based steam	Europe	1.56	0.458	0.081

a Boundary limits are cradle-to-gate (from extraction of resources to production of H_2_O_2_), no mass or economic allocation applied.

b Notes: Functional unit: 1 kg pure (100%) H_2_O_2_. LCIA Method: Recipe 2016 v1.09, midpoint (H). Data referring to the geographical area of Europe are retrieved from Ecoinvent version 3.10.1. system model cutoff (i.e., H_2_O_2_ production, product in 50% solution, chlor-alkali electrolysis, membrane cell).

### Safety

The AO process has historically been favored for its safety, as it enables the production of H_2_O_2_ via an indirect reaction of H_2_ with O_2_, making it suitable for large-scale production.^6,22^ However, the process presents inherent risks related to the substances used and the operating conditions. One major concern is the exothermic decomposition of the produced H_2_O_2_, which can be initiated by elevated temperatures or incompatible substances such as transition metals or reducing agents, and lead under certain conditions to thermal runaway.^171^ The decomposition rate is influenced by the H_2_O_2_ concentration, making precise control of solution composition and temperature critical, particularly during extraction, purification, or distillation steps.^172^ The highly exothermic hydrogenation and oxygenation reactions also pose thermal runaway risks. Explosive atmospheres are another safety challenge. H_2_ explosions may result from air/H_2_ mixtures or leaks near the hydrogenator and auxiliary equipment. During the oxygenation, extraction, and regeneration phases, flammable organic solvents (C9–C10 aromatics) present a risk of air-organic vapor explosions. Special attention to H_2_O_2_/H_2_O/organic solvent mixtures during H_2_O_2_ extraction is required, as explosion limits depend on the chemical composition and operating conditions.^173^ Similar risks have been identified in alternative auto-oxidation processes using other alcohols, such as isopropyl alcohol.^174^ Finally, large-scale production using the AO process implies storage and transportation of H_2_O_2_, stages that present additional risks of thermal runaway.

Due to its extensive and long-standing application, the risks associated with the AO process are well-documented and effectively managed. In contrast, the more recently developed alternative processes have less comprehensive hazard documentation. Nonetheless, a first identification of the potential risks associated with these emerging processes is proposed based on the currently available information.

DSHP is a promising alternative to the AO process but poses substantial safety challenges linked to explosive gas mixtures, secondary reactions, and flammable solvents. First, the triphasic reaction involves a mixture of gaseous H_2_ and O_2_ that reacts on a solid catalyst in a liquid solvent. Consequently, DSHP presents a risk of explosion when H_2_ and O_2_ are mixed at concentrations within the explosive range. Ensuring safety requires meticulous control of the H_2_:O_2_ ratio, typically maintaining H_2_ concentrations below 4 vol % or O_2_ concentrations below 6% to remain outside the explosive range (Figure S6). However, such conditions can limit the H_2_O_2_ productivity. Higher H_2_ and O_2_ concentrations within the flammability range enhance both productivity and selectivity but require special and complex reactors for safe operation.^175^

Second, side reactions, including H_2_ combustion, H_2_O_2_ decomposition, and H_2_O_2_ hydrogenation to H_2_O, further complicate the process.^6,22,176^ These exothermic secondary reactions reduce the overall efficiency and introduce additional safety hazards, particularly in systems where heat removal is challenging.^22^ The choice of catalyst is critical to inhibiting undesired side reactions. Finally, solvents, typically MeOH or other alcohols,^176^ facilitate gas dissolution, improving reaction efficiency.^22^ However, the high MeOH vapor pressure and flammability pose explosion risks, with incidents reported during experimental synthesis.^176^ Alternatives such as H_2_O-based systems have been proposed but may sacrifice efficiency.^6^

Innovative reactor designs aim to address these safety challenges. Membrane reactors physically separate H_2_ and O_2_, significantly reducing the risk of explosive mixtures. In these systems, H_2_ is supplied as atomic H via a dense palladium membrane, which also acts as a catalyst. Similarly, designs like microreactors allow safe operation within the flammability domain of the H_2_–O_2_ mixture.^177,178^ The small dimensions of such processes both facilitate contact between the gas mixture and the reactor wall and inhibit the spread of flame and explosion inside the equipment.^177^ Moreover, in the case of an explosion, less severe consequences are expected due to the small volumes and quantities operated. However, such systems remain complex and expensive for large-scale industrial deployment.^175^ The alternative synthesis route using CO, O_2_, and H_2_O has shown potential, with heterogeneous catalysts like Au/TiO_2_ achieving high H_2_O_2_ concentrations under mild conditions. While safer and suitable for integrated processes, this method is still in early development.^6^

The electrochemical synthesis of H_2_O_2_ has been extensively studied, yet safety considerations remain poorly addressed in the literature. The risks associated with this type of process vary widely, depending on the reactants, electrodes, electrolytes, and operating conditions involved. Fuel-cell electrochemical processes use H_2_ and O_2_ as reactants. The separation of gases by an electrolytic membrane minimizes the risk of explosive mixtures.^176,179^ However, the inherent hazards of handling H_2_, such as leakage and potential ignition, persist and require safety measures. Other electrochemical processes use H_2_O and O_2_ as reactants, providing a safer alternative.^179^ Commonly used electrolytes containing Na_2_SO_4_ ^80^ and Na_2_CO_3_ ^72^ entail minimal risk. On the contrary, strong acids such as HClO_4_ ^65^ and H_2_SO_4_ ^177^ present significant risks due to their corrosivity and potential for fast and exothermic reactions.

Finally, the electrochemical cell configurations can mitigate risks. For instance, flow cells designed to enhance mass transport reduce risks associated with H_2_O_2_ accumulation. Overaccumulation of H_2_O_2_ can lead to its decomposition, which generates heat and O_2_,^16^ increasing explosion and fire hazards. Another example is the development of SSE systems that replace liquid electrolytes and offer a promising route for safer and more efficient H_2_O_2_ production. SSEs eliminate the need for postsynthesis separation steps, reducing process complexity and associated risks.

Available information is even more limited for other emerging processes that are at the lab scale, such as BESs. The unequal level of development and the diversity of the new technologies explored herein do not allow a direct comparison of their safety performance, particularly on an industrial scale. Advances in catalysts and reactor designs are notably essential to mitigate the safety issues.

Plasma-assisted processes present the advantage of operating directly with an H_2_/O_2_ feed without the need for any catalyst or solvent. For safety reasons, early developments on such a process were conducted strictly below 6 mol % O_2_ ^131^ to stay out of the flammability domain. Moreover, the electric field in the plasma reactor can be an ignition source for an explosion. Therefore, the control of both the O_2_ content and the electric power represents important safety challenges^131^ that greatly limited the practicality of H_2_/O_2_ plasma methods in the past. To overcome these constraints, new optimized reactors were proposed for safer plasma production, allowing similar operations at higher concentrations, and a self-cooling-double discharge (SC-DDBD) reactor demonstrated the possibility to extend the concentration domain for safe use up to 30 mol % of O_2_ in the feed gas mixture.^132^ As an explanation, the authors proposed that H_2_/O_2_ explosions in a plasma process would be mainly due to the formation of H_2_O from OH and H_2_, and the generation of OH from reactive species (mainly radicals) is fast and highly exothermic. On the contrary, in the SC-DDBD reactor, the concentration of reactive species could be reduced thanks to more uniform and diffuse discharges. This demonstrates well the critical importance of safety for the development of a plasma process suitable for industrial applications.

### Challenges and Future Perspectives

While at present H_2_O_2_ production is dominated by the large-scale AO process, the drive toward more sustainable chemical processes with smaller CO_2_ footprints continues. Although the AO process is economic, it can clearly be improved from energy and climate perspectives. Utilities (electricity) and H_2_ production are the key sources of pollutants in the AO process. Increasing the share of renewable electricity into the AO process, either as utility (replacing fossil-fuel-based electricity and heat) or as the main energy source for raw material production (e.g., H_2_), is necessary for reducing the environmental burden of the AO process. Sourcing enough H_2_ via electrolysis to meet the production requirements of world-class AO plants has its own challenges. There are, in addition, efficiency and global sustainability questions related to the production of green H_2_.

The transition to electricity-driven alternative technologies for H_2_O_2_ production could attain lower CO_2_ footprints, considering that electrification will be coupled with the decarbonization of the grid electricity. However, the lack of demonstration at a larger scale is the current challenge to be overcome. Indeed, as fossil-fuel-based processes have an incumbency advantage from years of optimization, technological newcomers must offer other advantages that might offset higher costs, at least until these newcomers have had sufficient opportunity to optimize their processes near the extent fossil-fuel-based ones have. For example, while the DSHP reaction is aesthetically appealing, safety remains an inherent challenge since processes developed for the DSHP reaction must be keenly aware of the explosion potential of H_2_/O_2_ mixtures. The low acceptable concentrations of either one of the reactants limit H_2_O_2_ yields. While this can be avoided through the use of palladium-based membranes that provide atomic H rather than molecular H_2_, these approaches suffer from low reaction rates and are costly to scale up due to the expense of palladium.^176^

Despite the continuing optimization of catalysts, electrodes, and reactor configurations to achieve the electrosynthesis of H_2_O_2_ reported in the literature, relatively few reports have tested the electrochemical system on a practical industrial scale. Standard industrial operation requires high current density (>300 mA/cm^2^), low cell potential (<2 V), high selectivity toward H_2_O_2_ (>90%), long-term electrode stability (>1000 h), and relatively low material costs, all of which should be simultaneously achieved.^101^ Indeed, various modifications, including heteroatom doping and the addition of supports, have been studied to improve the stability and durability of the electrodes. However, electrode stabilities are generally still too low (up to 100 h) for industrial applications. Hydrophobic carbon GDEs exhibit excellent H_2_O_2_ activity and selectivity, but flooding of the GDE might occur.^99^ The electrode stability also faces a challenge under high current density for long-term operations.^89^

For the deployment of BESs for H_2_O_2_ production at larger production scales, the complexity of the process is still a major challenge for upscaling. Stacking multiple BES modules in series is an appropriate option,^111,180,181^ but expensive. However, due to the system overpotentials, 2–3 orders of magnitude lower H_2_O_2_ production rate at a pilot scale was reported primarily due to its decomposition, as compared to that obtained for lab-scale studies. Additionally, the low efficiency and reduced rate of H_2_O_2_ production further impede the upscaling of BESs.^182^ Therefore, it has been considered that BES production of H_2_O_2_ for in situ oxidation for environmental remediation would be more practical than direct H_2_O_2_ synthesis.^182^

Plasma-assisted H_2_O_2_ production may hold promise since, unlike other electrified technologies, plasma reactors and specifically DBD reactors are available for large-scale production. However, the poor energy efficiency, low yield, and high energy demand hinder wide industrial deployment. Minimization of the energy dissipation into the hardware (dielectric barrier) and selective boosting of the electron-impact reactions that effectively result in H_2_O_2_ formation may facilitate the commercialization of the technology. The use of an aqueous solution as a grounding electrode and cooling agent, removing resistive and reaction heat, has been suggested as one approach to improve the energy efficiency.^133^ While plasma-based production of H_2_O_2_ presents a cleaner, safer, and more flexible alternative to traditional methods, the low yield and high energy demand currently limit the application of plasma-assisted H_2_O_2_ at an industrial scale.

Although the upscaling of emerging H_2_O_2_ technologies is a necessary step to determine their long-term commercial prospects, large-scale production along the lines of the AO process may not be needed. Issues related to transportation (logistics), safety (intermediate storage), and opportunities to benefit from fluctuations in renewable electricity supply may change the production landscape. Since H_2_O_2_ is produced centrally and in large quantities, transport of the product to customers, large and small, is required. This is perhaps an under-appreciated climate change contribution from H_2_O_2_ production. Since H_2_O_2_ can be unstable, particularly for concentrated solutions, decomposition hazards must be addressed during transport and storage. Hence, H_2_O_2_ is generally diluted to 50% (or lower), ensuring that at least half of the transported weight is H_2_O.^183^ While the climate change aspect of the transport of diluted H_2_O_2_ can be mitigated by using trucks running on electricity or renewable fuels, there are other environmental and social aspects that must still be considered, such as wear and tear on the transportation infrastructure and risks associated with the transport of a very oxidizing chemical through cities and towns. Additionally, stabilizers (like sodium phosphate, phosphoric acid, or acetanilide) to reduce further decomposition and contamination hazards are added to marketed aqueous solutions of H_2_O_2_. These stabilizers must then be removed or otherwise accounted for by H_2_O_2_ end-users, which places extra demands on their processes. This extra handling decreases the overall energy efficiency and increases the climate footprint and cost of downstream applications.

Technologies with flexible capacities may serve the market better. Since electrified chemical production is less influenced by the economies of scale that have led to centralized chemical production process, a direct electrochemical production of H_2_O_2_ clearly has its advantages. Small and flexible H_2_O_2_ production facilities offer the potential of lower production cost by benefiting from periods when renewable electricity is abundantly available and cheap. This is of course under the precondition that operational savings compensate for the required investments in additional production and storage capacity. Such small, flexible facilities might additionally provide H_2_O_2_ locally to end-users, eliminating the need for long-term stabilization and transport and simplifying downstream production processes, as long as the desired H_2_O_2_ concentrations for the intended applications can be achieved. An advantage of modular, on-site, and on-demand H_2_O_2_ production might be the reduction of concerns about H_2_O_2_ decomposition, shipping, and the use of stabilizers and the ability to provide exact H_2_O_2_ concentrations as needed. All of these qualities will simplify downstream applications and improve their environmental and economic profiles.

Regardless of whether renewable electricity is integrated into the AO process or used to establish new H_2_O_2_ production technologies, the intermittency and geographic availability of renewable energy supplies that can be used to power electrified H_2_O_2_ production technologies will also affect production trends and subsequently dictate the features of the new technologies. For the use of renewable energy in industrial electrochemical plants, the process must be able to operate dynamically and in discontinuous mode. This dynamic operation is very challenging, as excessive dynamic operation (variable power) and frequent starts and stops may affect the efficiency and stability of the process and may decrease the material lifetimes. To improve this situation, the degree of robustness and the selectivity of any electrochemical reaction need to be addressed. Especially, the anode reaction has to tolerate a wide range of current densities. The cathode has to be tuned to optimize energy efficiency and downstream integration. At low current densities, the radical density might be low, and the formation of peroxo-compounds may not be efficient. If the current density is too high, a high gas fraction in the electrolyte will introduce mass transport limitations and, therefore, lead to the oxidation of H_2_O and O_2_ formation. Special attention has to be paid to the cell design to handle the variety of gas fraction ratios.

Future research and development efforts for electricity-driven and on-demand H_2_O_2_ production processes should focus on addressing these critical remaining issues.

- Design and synthesis of high-performance and stable electrocatalysts for both anode and cathode: a comprehensive understanding of the involved reaction mechanisms and catalyst structure is required to increase the selectivity toward H_2_O_2_ production and ensure stable performance.
- Superior electrochemical cell/reactor design: improved cell/reactor design can enable scalable, robust, and renewable energy integrated H_2_O_2_ production.
- Demonstration at a higher scale: all of the emerging H_2_O_2_ production processes are currently at lab scale. Demonstration at a larger scale (i.e., pilot scale) is needed to gain hands-on experience on technical and integration challenges that might arise. Bridging the gap between lab- and large-scale applications will also be able to showcase the feasibility of the emerging processes.
- Product purification: current research efforts overlook H_2_O_2_ purification from byproducts or from liquid electrolytes. However, as the downstream product purification process steps depend on the targeted application of the produced H_2_O_2_ and can be the cost driver for large-scale production systems, they should be carefully considered.
- Minimization of the environmental impact across the entire electricity-driven H_2_O_2_ production process: key efforts should target reducing carbon emissions through increasing the penetration of renewable electricity and improving overall energy efficiency. Additionally, optimization of the system design to eliminate hazardous byproducts and to minimize resource consumption will be essential to achieve a truly sustainable, low-carbon H_2_O_2_ production pathway. Comprehensive LCA studies focused on fully electrified emerging technologies for H_2_O_2_ production are needed.
- Improved investigations of safety issues for alternative processes: analyses of these processes and their modular integration for on-demand production are needed to enable comprehensive risk management strategies, which are crucial for successful implementation at user sites. These must account for full integration at end-user sites, including, notably, substance storage and operator training, especially in industries unfamiliar with chemical synthesis.

In conclusion, it is hoped that this review of alternative H_2_O_2_ production processes has at least raised awareness of some of the technical, techno-economic, life cycle, and safety challenges that these technologies have to solve in order to compete with the entrenched AO process. As many of these challenges cannot be properly addressed based solely on lab-scale technology developments, efforts to scale up some of the more promising technologies and better evaluate their industrial fitness are required. For electrified solutions, the overall challenge above and beyond the pure technical challenges is one of infrastructure. Technologies reliant on renewable electricity generation and storage, necessary for any modular, “on-demand” H_2_O_2_ production process, must not be a slave to the inherent fickleness of renewable energy production. Grid stabilities that can tolerate the electricity supply fluctuations as increased amounts of renewable electricity enter the grid are also critical to the eventual success of electrified technologies. Furthermore, as more and more sectors of our society become electrified, the overall availability of renewable electrical energy and its best deployment between sectors for achieving the greatest reduction of society’s CO_2_ footprint must be addressed.

## Supplementary Material



## Footnotes

a One EUR = 0.92 USD.

## Notes

Figures of plasma reactors and processes, tables of recalculated values and various operational parameters for the techno-economic evaluations, figures of GWP(100) breakdowns, the variation of electricity source on the H_2_O_2_ production footprint, and the explosion limits of H_2_/O_2_ mixtures (PDF)
