# Supplementary material for: Alternative H2O2 Production Processes: An Outlook on Candidate Technologies Beyond the Anthraquinone Process
Source: ACS Omega. 2025 Dec 10;10(50):61076–95. doi: 10.1021/acsomega.5c07503 (PMC12750225; doi:10.1021/acsomega.5c07503)
Supplement: Supplementary file 1 [file ao5c07503_si_001.pdf]

Supplementary Information

Alternative H_2O_2 production processes: an outlook on candidate technologies beyond the anthraquinone process

Stavros-Alexandros Theofanidis†, Amvrosios G. Georgiadis†, Christianus J.W. Hop‡, Xiaobin Yu‡, Vasileia-Loukia Yfanti†, Guillaume Fayet§, Claire Villemur§, Hank Vleeming‡*, Evangelos Delikonstantis†* and Richard H. Heyn#*

† AristEng S.à r.l., 77, Rue de Merl, L-2146, Luxembourg City, Luxembourg

‡ Process Design Center BV, Paardeweide 7, NL-4824EH Breda, The Netherlands

§ Ineris, Parc Technologique ALATA, BP 2, 60550 Verneuil-en-Halatte, France

# SINTEF Industry, P. O. Box 124 Blindern, 0314 Oslo, Norway.

Corresponding Authors

Hank Vleeming, vleeming@process-design-center.com, + 31 76 5301 906

Evangelos Delikonstantis, evangelos.delikonstantis@aristeng.lu, + 352 661 213 742

Richard H. Heyn, rhh@sintef.no, +47 982 43 927

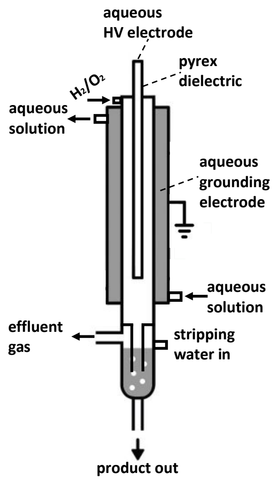

Figure S1: Schematic structure of the DBD reactor with double aqueous electrodes, used with permission from JohnWiley & Sons - Books.[131]

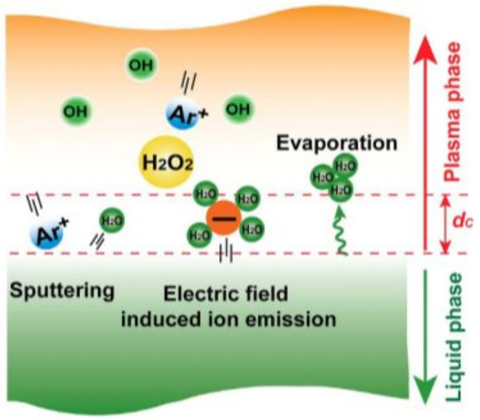

Figure S2: Three main processes occurring at the plasma liquid interface, used with permission from Springer Nature BV.[145]

Table S1: Reported and recalculated cost comparison for an electrosynthetic H_2O_2 process.

		**Reported**[155]	**Recalculated**
Capital cost (EUR/ton)		121	1103
Operational cost (EUR/ton)	Feed streams	14	811
	Electricity	305	375
	Others	103	2247
Manufacturing cost (EUR/ton)		543	4536

Table S2. A list of various operational parameters for techno-economic evaluation.

Unit	Operational parameters	Value	Source
Electrolyzer	Applied voltage	2.5 V	[150]
	Current density	300 mA/cm^2	[150]
	Electrolyte	Na_2SO_4 (1M)	[150]
	Electrode area (lab)	1 cm^2	[150]
	Electrode area (designed)	1120 cm^2	Assumed
	Electrolyte circulation speed	2 mL/min	[150]
	Current efficiency (anode)	100%	Assumed
	O_2 recovery (anode)	75%	[150]
	Current efficiency (cathode)	92%	[150]
	O_2 utilization efficiency	25%	Assumed
	Electrolyzer cost	1288 EUR/kW (1400 USD/kW)	[150]
	Electrode/membrane cost	2760 EUR/m^2 (3000 USD/m^2)	[150]
	Electrode/membrane cost life span	3 years	Assumed
Stripper	H_2O_2 entering stripper	4.6 wt%	[150]
Rectifier	H_2O_2 entering rectifier	2.0 wt%	US3694154 Patent
	H_2O_2 leaving rectifier	35 wt%	US3694154 Patent
Others	Na_2SO_4 recovery	75%	[150]
	Plant capacity	25 t/d	Assumed
	Depreciation time	10 years	Assumed
	Electricity (RES) price	50 EUR/MWh	Assumed
	Na2SO4 price	250 EUR/ton Na_2SO_4	Chemanalyst1
	O_2 price	270 EUR/ton O_2	Chemanalyst2
	Deionized water price	1.5 EUR/ton H_2O	Assumed

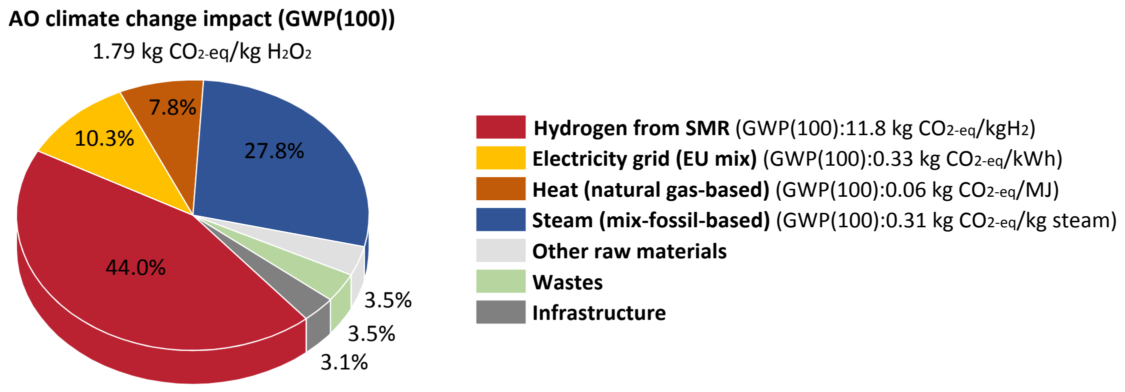

Figure S3: Percentage breakdown of raw materials, utilities, waste and infrastructure contributions to the global warming potential midpoint (GWP (100)) impact of AO process (1.79 kg $CO_{2\text{-eq}}/kg_{H2O2}$). Data retrieved from Ecoinvent database.[159]

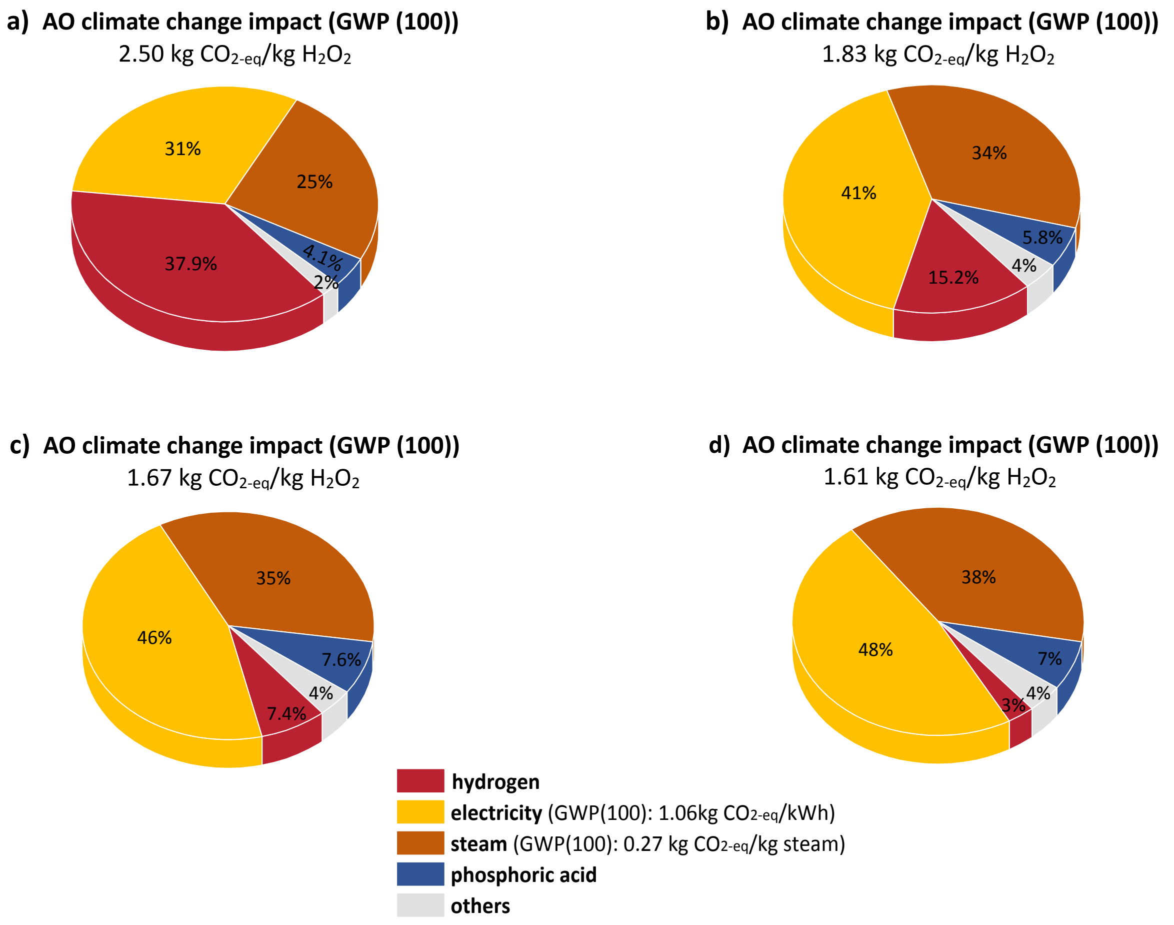

Figure S4: Carbon footprint breakdown and benchmarking of H_2O_2 production via the AO process, using various H_2 sources: a) SMR, b) coal coking and gasification c) hydrocarbon cracking, and d) chloro-alkali electrolysis.[158]

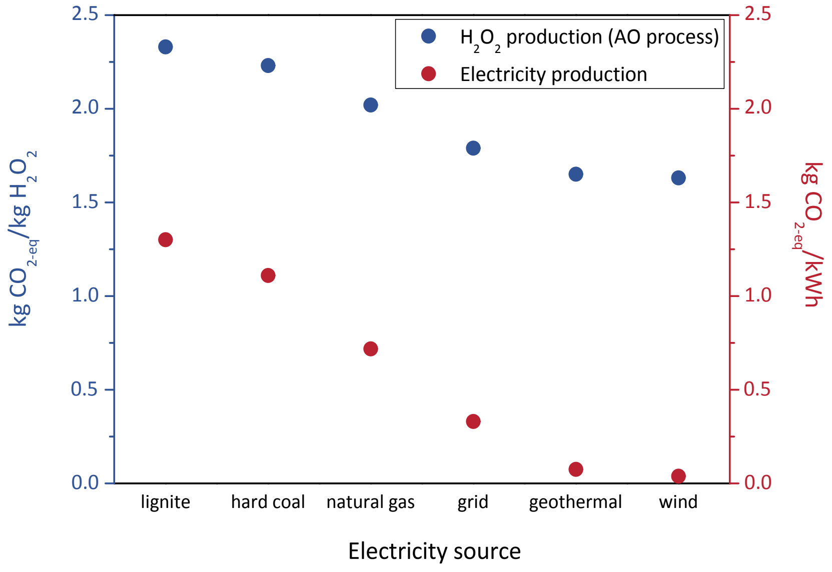

Figure S5: Variation of H_2O_2 and electricity production process carbon footprint ($kg_{CO2\text{-}eq}/kg_{H2O2}$) as a function of electricity source (lignite, hard coal, natural gas, market group of EU grid electricity, geothermal, wind). All the LCI data for the electricity production and the data for the AO process are retrieved from the Ecoinvent database[166] and are the same with those presented above (Figure S3).

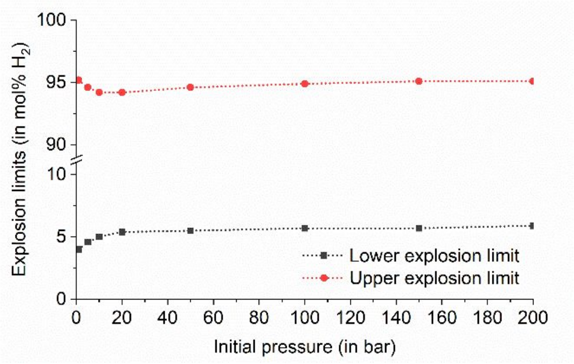

Figure S6: Explosion limits of H_2-O_2 mixtures based on experimental data measured at room temperature with different initial pressures.[184]

References (numbers are the same as in the main text).

[131] Yi, Y.; Zhou, J.; Guo, H.; Zhao, J.; Su, J.; Wang, L.; Wang, X.; Gong, W. Safe Direct Synthesis of High Purity H_2O_2 through a H_2/O_2 Plasma Reaction. *Angew. Chem. Int. Ed.* **2013**, *52*, 8446–8449. https://doi.org/10.1002/ANIE.201304134.

[145] Liu, J.; He, B.; Chen, Q.; Li, J.; Xiong, Q.; Yue, G.; Zhang, X.; Yang, S.; Liu, H.; Liu, Q. H. Direct Synthesis of Hydrogen Peroxide from Plasma-Water Interactions. *Sci. Rep.* **2016**, *6*, 38454. https://doi.org/10.1038/srep38454.

[156] Lee, B. H.; Shin, H.; Rasouli, A. S.; Choubisa, H.; Ou, P.; Dorakhan, R.; Grigioni, I.; Lee, G.; Shirzadi, E.; Miao, R. K.; Wicks, J.; Park, S.; Lee, H. S.; Zhang, J.; Chen, Y.; Chen, Z.; Sinton, D.; Hyeon, T.; Sung, Y. E.; Sargent, E. H. Supramolecular Tuning of Supported Metal Phthalocyanine Catalysts for Hydrogen Peroxide Electrosynthesis. *Nat. Catal.* **2023**, *6*, 234–243. https://doi.org/10.1038/s41929-023-00924-5.

[165] Jia, Y.; Bai, Y.; Chang, J.; Zhai, Y.; Zhang, T.; Ren, K.; Hong, J. Life Cycle Assessment of Hydrogen Peroxide Produced from Mainstream Hydrogen Sources in China. *J. Clean. Prod.* **2022**, *352*, 131655. https://doi.org/10.1016/J.JCLEPRO.2022.131655

[166] ecoinvent. ecoinvent. https://ecoinvent.org/.

[184] Schroeder, V. & Holtappels, K. Explosion Characteristics of Hydrogen-Air and Hydrogen-Oxygen Mixtures at Elevated Pressures. *Int. Conf. Hydrog. Saf.* Paper No. 120001 (2005).

US3694154 Patent: W. S. Harper, D. W. Daigler, and E. Aurora, “Production of Hydrogen peroxide from aqueous acidic solution obtained by hydrolysis." 1968.

Chemanalyst1: Chemanalyst, “Track Sodium Sulphate Price Trend and Forecast In Top 10 Leading Countries Worldwide.” 2024.

Chemanalyst2: https://www.chemanalyst.com/Pricing-data/oxygen-1575
